# Novel protocatechuic acid encapsulated bovine serum albumin functionalized folic acid nanoparticles for targeted therapy in urethane-induced lung cancer model

**DOI:** 10.1038/s41598-025-08465-6

**Published:** 2025-07-16

**Authors:** Alaa I. Ibrahim, Heba A. Sahyon, Adel M. Attia, Ashraf A. El-Shehawy

**Affiliations:** Faculty of Science, Chemistry Department, Kafrelsheikh University, El Giesh Street, Kafrelsheikh, 33516 Egypt

**Keywords:** Protocatechuic acid, Albumin nanoparticles, CD44, NF-kB, FAK, MAPK, Biochemistry, Cancer

## Abstract

Lung cancer mortality rates are rising globally, increasing the need for innovative, natural compounds with fewer side effects. Protocatechuic acid (PCA) is a natural phenolic compound with cytotoxic effects against human lung cancer cells, but its poor water solubility limits its use. We hypothesized that encapsulating PCA within bovine serum albumin (BSA) nanoparticles would enhance its solubility compared to previous PCA-loaded-nanocarriers. So, we encapsulated PCA within BSA-nanoparticles conjugated with folic acid (FA) and evaluated its targeting efficacy in lung cancer cell line and lung cancer mouse model. The resulting nanocomposite (PCA-BSA@FA-NPs) had a 229 nm size. *Our in vitro* study indicated that PCA-BSA@FA-NPs demonstrated a 92.03% reduction in toxicity compared to doxorubicin, and a 59.67% reduction compared to PCA when tested against normal WI38 cells. In the lung cancer mouse model, the NF-κB expression decreased by 179% in the PCA-BSA@FA-NPs-treated group compared to the PCA-treated group. Both groups showed significant reductions in MAPK and FAK, with greater declines of 172% for MAPK and 316% for FAK in the PCA-BSA@FA-NPs-treated group. In conclusion, PCA-BSA@FA-NPs may serve as a promising therapy for targeting lung cancer with reduced toxicity.

**Supplementary Information:**

The online version contains supplementary material available at 10.1038/s41598-025-08465-6.

## Introduction

Lung cancer ranks as the second most prevalent form of cancer and is a major contributor to cancer-related mortality worldwide, with non-small cell lung cancer (NSCLC) making up 80% of cases^1^. Treatment options for NSCLC include surgery, chemotherapy, immunotherapy, and radiotherapy, depending on the cancer’s stage and other factors^2^. For patients who are unable to undergo surgery due to specific clinical circumstances, noninvasive therapies may offer a viable option. However, the effectiveness of these noninvasive treatments can be limited by toxicity to vital organs or tumor resistance, which can significantly affect both the quality of life and treatment outcomes for patients^3^.

Protocatechuic acid (PCA) is a natural phenolic compound found in blackberries, black currants, cauliflower, and lentils. PCA is known for its cytotoxic effects on human lung cancer cell lines, as well as its antioxidant properties^4^. Several studies indicate that PCA is cytotoxic against several in vitro human cancer cell lines^5^. Its anticancer effects are attributed to mechanisms such as plasma membrane destruction, infiltration into cancer cells, and the induction of apoptosis, which together lead to the inhibition of cancer cell growth^6^. PCA’s poor solubility in water limits its application in the biological field, particularly in cancer treatment^7^. In a previous study, PCA was loaded onto chitosan nanoparticles to enhance their therapeutic efficacy against human lung cancer cells and improve their antioxidant activity^8^. However, the poor solubility of chitosan poses an obstacle to its potential application as a drug carrier, as chitosan nanoparticles are poorly soluble in water and dissolve only in organic solvents^9^. Therefore, the researchers turned to modified chitosan nanoparticles, which enhanced the efficacy of PCA by improving their delivery capacity and amplifying their toxic effects against human lung cancer cells. This leads to more effective anticancer responses^9^. From our perspective, albumin would be a better alternative to chitosan. Albumin is a natural polymer produced by the body, while chitosan is also a natural polymer, but is not produced within the body.

Bovine serum albumin nanoparticles (BSA-NPs) present a promising alternative to chitosan nanoparticles due to their high solubility in water. BSA-NPs contain charged amino acids that non-covalently serve as transport systems for various phenolic acids, such as caffeic acid and salicylic acid, facilitating their entry into cells. The surface properties of BSA-NPs, including charge, binding sites, and amino acid composition, enhance their ability to bind to phenolic acids^10^. Furthermore, electrostatic adsorption of phenolic acids on the BSA surface is more effective when the BSA molecules are smaller, thereby improving drug distribution control^11^.

Recently, several researchers have focused on folate receptors that target malignant tumors, which are overexpressed in cancer cell membranes while being at low levels in normal tissues. Folic acid (FA) possesses several advantageous properties that make it ideal for drug delivery: it is biocompatible, non-immunogenic, stable, and safe. Additionally, it plays a critical role in DNA biosynthesis and cell proliferation^12,13^.

Inflammatory signaling pathways include nuclear factor kappa B (NF-kB), and mitogen-activated protein kinases (MAPKs) are often involved in the survival and proliferation of lung cancer cells. NF-κB plays a role in the proliferation of lung cancer by preventing programmed cell death^14^. MAPKs are also capable of influencing NF-κB activity, leading to a feedback loop that enhances oncogenic signaling in lung cancer. MAPK pathways can also be stimulated by focal adhesion kinase (FAK), which subsequently boosts NF-κB signaling, establishing a network that facilitates tumor growth and metastasis. This interaction implies that targeting these pathways together might offer a more effective treatment strategy for lung cancer, especially in addressing resistance to current therapies^15^. We hypothesize that encapsulating PCA within BSA-NPs and conjugating these nanoparticles with folic acid will enhance PCA’s efficacy against lung cancer. Furthermore, this nanocomposite will be administered orally to evaluate its potential as a noninvasive therapy for lung cancer in a mouse model.

In this study, we aim to encapsulate PCA within BSA-NPs and conjugate these nanoparticles with folic acid to target lung cancer cells through dual mechanisms: folate receptor-mediated endocytosis and albumin binding. The resulting nanocomposite will be characterized, and its in vitro effects will be evaluated against a human lung cancer cell line, A549, and compared with doxorubicin, a standard anticancer drug. The antioxidant activity of the resulting nanocomposite will be assessed using the DPPH (free radical scavenging) assay. Additionally, we will investigate the in vivo anticancer effects of the oral administration of the nanocomposite and PCA using a lung cancer mouse model.

## Materials and methods

### Chemical

Protocatechuic acid (PCA) was purchased from Xi’an Huilin Biotech. Co., Ltd. in China (CAS: 99-50-3; 98% specific). Bovine serum albumin (purity 98%, molecular weight 69.323 kDa, CAS number A3059), aqueous glutaraldehyde (24%), and absolute ethanol (99%) were acquired from Sigma-Aldrich. Human lung cancer cell line (A549) and normal human lung fibroblasts (WI38) were obtained from the American Type Culture Collection (ATCC, Rockville, MD); fetal bovine serum, RPMI-1640, HEPES buffer solution, L-glutamine, gentamycin, and 0.25% trypsin-EDTA were purchased from Lonza (Belgium); and dimethyl sulfoxide (DMSO), MTT, and trypan blue dye were purchased from Sigma (St. Louis, Mo., USA). Ascorbic acid, methanol (99%), and 2,2-diphenyl-1-picrylhydrazyl (DPPH) were acquired from Sigma Aldrich, USA. The catalase kit was purchased from Egypt’s Biodiagnostic. Sigma-Aldrich Canada Ltd. provided the phosphate buffer (0.1 M, pH 8.3), nicotinamide adenine dinucleotide (NAD), nitro tetrazolium blue (NBT) 0.3 mM, and phenazine methosulphate (PMS) 0.93 mM, phosphate buffer saline (pBS), trichloroacetic acid (TCA) 30%, ethylene diamin tetra acetic acid EDTA, and thiobarbituric acid TBA (1%). Urethane (97%) was obtained from Thermo Fisher Scientific. Albumin, creatinine, glutamic pyruvate transaminase (SGPT), and serum glutamic oxaloacetic transaminase (SGOT) were measured by BioMed reagents acquired from Egy.CHEM. for PCR RNA extraction by the Superscript IV One-Step PCR kit the Superscript IV One-Step PCR kit (Cat# R2072, ZYMO RESEARCH CORP, USA) and the Superscript IV One-Step PCR kit (Cat# 12594100, Thermo Fisher Scientific, Waltham, MA, USA). All chemicals were of high analytical grade to ensure their purity and accuracy.

### Preparation of BSA-NPs

The desolvation technique is the simplest method for the preparation of BSA-NPs^16,17^in which BSA (100 mg) was dissolved in deionized water (2 mL) at pH 7.4 and then agitated for 15 min at room temperature (25 °C) at 700 rpm. With continuous stirring, ethanol was added by dropping at a rate of 1 mL/min to the dissolved BSA until it was transformed into NPs, where the formation of a turbid suspension indicates the formation of NPs. After 15 min, 10 µL of 24% (v/v) glutaraldehyde aqueous was added to bind the dissolved BSA NPs, with continuous stirring overnight. After stirring, the NP suspension was purified by undergoing three cycles of centrifugation at 12,000 rpm (Hermle Labortechnik GmbH) for 20 min to remove undissolved BSA, glutaraldehyde, and ethanol. After each centrifugation step, the NPs were resuspended in 5 mL of deionized water using a Germany Elmasonic P60H ultrasonic bath for 5 min. The samples were then dried using a Martin Christ lyophilizer.

### Preparation of PCA-encapsulated BSA-NP (PCA-BSA-NPs)

The method used to prepare nanoparticles from bovine serum albumin and load PCA onto them involves a specific strategy due to PCA’s lack of water solubility. First, PCA is dissolved in ethanol. This ethanol solution is then added dropwise to the dissolved bovine serum albumin, resulting in the formation of a nano compound^17^. The final solvent-to-antisolvent ratio in the formulation was 1:1 v/v. The BSA solution, consisting of 100 mg of BSA powder dissolved in 2 mL of deionized water with a pH of 7.4, was stirred at 700 rpm at room temperature for 15 min. Then, 20 mg of PCA was dissolved in 2 mL of ethanol and dropped continuously from a syringe pump at a rate of 1 ml/min as a dissolving agent. While PCA exhibits higher solubility in ethanol, the rapid addition of the ethanol-containing solution to the antisolvent phase (BSA dissolved in water) leads to a sudden decrease in the solubility of both the nanoparticle matrix material (BSA) and PCA. This rapid supersaturation drives the immediate precipitation and nucleation of the nanoparticles, effectively trapping PCA within the forming solid matrix before it can diffuse away into the antisolvent. As a result, a specular suspension was obtained, indicating the formation of nanoparticles. Furthermore, PCA may exhibit hydrogen bonding with the BSA matrix, which further promotes its incorporation and retention within the nanoparticle structure during the precipitation process. The obtained nanoparticles were stabilized with 10 µL of 24% glutaraldehyde^18^. Then, the nanoparticles were centrifuged three times (12,000 rpm, 20 min) to remove the non-dissolved albumin, excess cross-linking agent, and organic solvent. The first centrifugation supernatant was used for the determination of non-desolvated albumin. Each centrifugation was followed by washing the powder with deionized water and throwing away the supernatant solution. The NPs were then redispersed in 5 mL of deionized water, and the solution was sonicated with ultrasound for 5 min. Finally, the product was dried in a freeze-dryer for 24 h at 55 °C and then incubated at 4 °C.

### Preparation of PCA-encapsulated BSA-NP conjugated folic acid (PCA-BSA@FA-NPs)

BSA (100 mg) was dissolved in 2 mL of deionized water, pH 7.4, and stirred at 700 rpm for 15 min to form a solution. Then, 20 mg of PCA was dissolved in 1 mL of ethanol and continuously dropped from a syringe pump at a rate of 1 mL/min as a solubilizing agent, and the carboxyl group of folic acid was then activated by carbodiimide reaction^19^. NaOH (1 N) was used to dissolve and activate FA, then 1 mL of activated FA solution was slowly added to the PCA-BSA-NPs solution and stirred for 30 min, then 10 µL of 24% (v/v) aqueous glutaraldehyde was added. This reaction was kept overnight in the dark with constant stirring at room temperature. The resulting compound was purified in the same way as the previous one.

### Characterization techniques

The formed NP composite powders were redistributed in deionized water and sonicated for 5 min at room temperature to ensure that the NPs were evenly distributed to ensure accuracy in the measurements. Each measurement was performed three times. Zetasizer Nano and He-Ne lasers at 633 nm were used to determine dynamic light scattering and zeta potential (DLS) to characterize BSA-NPs, PCA-BSA-NPs, and PCA-BSA@FA-NPs. JEOL, JSM-IT 100 Scanning electron microscopy (SEM) characterization is used to determine the morphology and structure of nanoparticles. The Jasco V-770 UV-visible spectrophotometer was used as it is characterized by accuracy and reliability in analyzing NPs in aqueous solutions through careful scanning of the wavelength range. The functional groups were identified, and any changes in the molecular structure were detected using Fourier transform infrared (FTIR) spectroscopy (JASCO, Tokyo, Japan, Model No. AUP1200343). For XRD analysis, the diffractometer (XRD 6100) was operated at a wavelength of 1.54056 Å, corresponding to CuKa1 radiation. This specific wavelength is commonly used due to its ability to interact effectively with the crystalline lattice structure of the sample under study. The physicochemical properties of the nanocomposites were analyzed using^1^H NMR spectroscopy. The proton NMR spectra of the products were recorded on a 500 MHz NMR spectrophotometer in DMSO.

### Determination of the loading and entrapment efficiency of PCA onto BSA-NPs

To evaluate the loading efficiency of PCA into PCA-BSA@FA-NPs, the drug loading ratio was evaluated. The final lyophilized PCA-BSA@FA-NPs (4 mg) were suspended in 3 mL of ethanol, followed by sonication for 15 min. Samples were placed in a shaker incubator and shaken at 37 °C overnight. After incubation, samples were taken and centrifuged at 20,000 rpm for 30 min to allow the nanocarriers to settle. The supernatant was collected and diluted. Then, using UV spectroscopy (Jasco V-770) at 254 nm, the PCA content was measured^20^. The measurement was performed at least three times. The formula for drug loading (DL) was as follows:

Loading capacity (%) = (weight of the total PCA - weight of PCA in the supernatant) / weight of the total PCA × 100.

Entrapment Efficiency (EE%) = (Total amount of drug used /Amount of drug entrapped​) ×100.

### In vitro drug release study

The in vitro drug release tests were performed to confirm the optimal release of PCA from the NPs. This applies to both physiological and cancer environments to confirm the efficacy of the resulting nanocomposite. PBS (phosphate-buffered saline) was used to redisperse 20 mg of NPs at pH 6.5 and 7.4, which correspond to cancerous and physiological environments, respectively. Then, they were placed individually in individual dialysis bags (molecular weight: 12,000–14,000 Da) and immersed in 40 mL of PBS at 37 °C with continuous stirring at 100 rpm. To measure the quantity of PCA released at various intervals during the experiment, 1 mL was aspirated from each baker at different intervals of time (0, 1, 2, 3, 4, 24, and 48 h). Meanwhile, after each aspiration, the baker’s solution was replaced with the same volume of fresh PBS. The absorbance of PCA was determined at 225 nm using a UV spectrophotometer, and three replicate measurements were made. To analyze the results, cumulative drug release over time was plotted^21^.

### In vitro cytotoxicity study

PCA, PCA-BSA-NPs, and PCA-BSA@FA-NPs were tested against A549 and WI38 human cell lines. The MTT test has been used to determine and compare the inhibitory effects of these substances on the growth of cancer cells in vitro and compare them to the standard anticancer drug (doxorubicin). The MTT test is mainly based on the conversion of the yellow color of tetrazolium bromide to a dark blue formazan derivative by mitochondrial succinate dehydrogenase in viable cells. Cell strains were cultured in RPMI-1640 medium supplemented with 10% fetal bovine serum. The antibiotics penicillin (100 units/mL) and streptomycin (100 µg/mL) were added to the culture media and incubated at 37 °C with 5% CO_2_. Using a 96-well plate, cell lines were cultured and incubated at 37 °C for 48 h under 5% CO_2_. After incubation, different concentrations (100–1.56 µg/mL) of PCA, PCA-BSA-NPs, and PCA-BSA@FA-NPs were added to treat cancer cells and then incubated for 24 h. Then, 20 µL of MTT (5 mg/mL) solution was added and incubated for 4 h. dimethyl sulfoxide (DMSO) 100 µL was added to each well to dissolve the formazan, producing purple-colored solutions. Then the colorimetric values at an absorbance of 570 nm were measured. Finally, IC_50_ values were evaluated and matched with those of PCA and doxorubicin (DOX). All experiments were performed in triplicate^22^.

### DPPH assay (2, 2-diphenyl-1-picrylhydrazyl)

The ability of the compounds to scavenge free radicals was evaluated in vitro using the DPPH technique^23^. DPPH stock solution (0.1 mM) was prepared, in which 39.4 mg of DPPH was dissolved in 1 mL of methanol and diluted with ethanol to achieve absorption of approximately 1 ± 0.2 at 517 nm using Jasco V-770 UV-visible spectrophotometer. Different concentrations of PCA, PCA-BSA-NPs, and PCA-BSA@FA-NPs (0.02, 0.04, 0.06, 0.08, and 0.1 mg/mL dissolved in methanol) were prepared and compared to the same concentrations of standard vitamin C. DPPH solution (0.1 mM) 2.9 mL was mixed with 0.1 mL of the sample or the standard at each concentration, and the mixture was then incubated for 30 min under dark conditions. Methanol (0.1 mL) was added in the blank tube instead of the sample. The absorbance of the samples and the standard was measured at 517 nm, and the absorbance of the blank tube was subtracted from the samples. Each analysis was carried out three times. The following equation was used to calculate the percentage of the DPPH radical inhibition:

% DPPH inhibition = ((A_blank_-A_sample_)/A_blank_) × 100.

### Superoxide dismutase (SOD) like activity and catalytic activity

SOD-like activity (scavenging activity) and catalytic activity were assessed as listed in our former study^24^.

### Experimental animals

Five-week-old male albino mice (weighing 33.125 ± 2.5 g) were used in our study. They were obtained from the animal facility of the Department of Chemistry at Kafrelsheikh University. Mice were housed in appropriate cages at approximately 25 °C. Each mouse received a constant diet consisting of food pellets and unlimited access to water. All methods and procedures described herein comply with the ARRIVE guidelines for reporting animal research. Ethical approval for the study was granted by the KFS-IACUC committee, approval number (No. KFS-IACUC/197/2024). All methods were carried out in accordance with relevant guidelines and regulations.

### Determining LD_50_ for PCA-BSA@FA-NPs

Male albino mice that had been fasted overnight were weighed and randomly divided into eight groups (five mice each). The nanocomposite (PCA-BSA@FA-NPs) was dissolved in sterile distilled water and vortexed well until dispersion. Eight groups of mice were orally injected with different PCA-BSA@FA-NPs doses (50, 100, 200, 500, 1000, and 2000 mg/kg) and observed for 24 h. The LD_50_ was determined based on animal mortality after 24 h.

### Experimental design

Healthy control mice were divided into four groups, each containing five mice. The healthy control group received daily IP injections with phosphate-buffered saline for 14 days. The PCA-control group received oral doses of 50 mg/kg of PCA dissolved in corn oil for 14 days^25^. In the third and fourth healthy groups, one group received a dose of 50 mg/kg body weight of PCA-BSA@FA-NPs dissolved in distilled water, equivalent to a high dose of PCA. The other group was administered 25 mg/kg body weight of PCA-BSA@FA-NPs, representing half the dose of PCA. Both treatments were given for 14 days and designated as high-dose PCA-BSA@FA-NPs and low-dose PCA-BSA@FA-NPs, respectively.

### Induction of lung cancer in a mouse model

Twenty adult male albino mice (30 g in weight) were given weekly intraperitoneal injections of fresh urethane in phosphate-buffered saline (pH 7) at a dose of 1 mg/g body weight for twelve weeks to establish a lung cancer model. After confirming the development of lung cancer in the thirteenth week, the urethane-induced mice were divided into four groups (five mice each): the positive control group, which was left without treatment. The PCA-treated group received 50 mg/kg body weight of PCA orally, dissolved in corn oil, for 14 consecutive days. The last two groups treated with PCA-BSA@FA-NPs were further divided into two subgroups: one subgroup received an oral dose of 50 mg/kg, while the other subgroup received 25 mg/kg for 14 days. These were referred to as the high-dose PCA-BSA@FA-NPs-treated group and the low-dose PCA-BSA@FA-NPs-treated group. All the animals were weighed and anesthetized at the end of the treatment period with intraperitoneal urethane (2 mg/g BW). KFS-IACUC committee’s procedures mandated that all mice be kept isolated from witnessing injection processes, blood collection, or organ harvesting to minimize their stress before anesthesia. The veterinary anesthesiologists from the KFS-IACUC committee outlined the anesthesia protocols and timing based on the mice’s sex, species, and age. The veterinarian noted that CO_2_ inhalation could damage the lungs, leading to hemorrhage, congestion, and emphysema, which could influence histopathological findings^26^. Consequently, they recommended the use of an intraperitoneal injection of urethane at a dosage of 2 mg/g of body weight for anesthetizing the mice^27^, followed by a fifteen-minute observation period to ensure they no longer responded to external stimuli, indicating that the anesthesia was successfully completed^28^. Anesthesia lasts six to eight hours. As a result, this technique was implemented to anesthetize all mice; afterwards, a heart puncture was performed to gather blood. Then, the blood was drawn from the heart by puncture, the rib cage was opened, and the lung was removed. Finally, all the dead mice are placed in a plastic waste bag and then delivered to the incinerator. The serum was separated by centrifugation for 10 min at 4000 rpm. Thereafter, samples were stored at -20 °C until testing. All mice’s lungs were removed, washed with physiological saline, dried on filter paper, and divided into four parts. One part was embedded in 10% formalin for histopathology and immunohistochemistry examination. Two parts were well rinsed in sterile PBS and then preserved at -20 °C for PCR testing and flow cytometry. The last part was left on filter paper to remove the excess PBS and blood, then weighed for the antioxidant enzymes’ determination. Lungs from each group were photographed, and the images were analyzed and quantified using ImageJ software (https://imagej.net/ij/) to determine the tumor area (%) and the percentage reduction of tumor area in the treated mice compared to the positive control group.

### Biochemical markers

BioMed kits were used to kinetically measure liver functions (blood glutamic pyruvic transaminase (GPT), glutamic oxaloacetic transaminase (GOT), and albumin and kidney function (creatinine).

### Determination of in vivo antioxidant enzymes

Frozen lung tissue (1 g) was homogenized in 9 mL of 0.01 M phosphate buffer at pH 7.4, then centrifuged at 2500 rpm for 15 min. The resulting supernatant was used for in vivo antioxidant and malondialdehyde (MDA) determination.

#### Superoxide dismutase enzyme assay

SOD was determined by the method of DeChatelet et al. (1974)^29^. The supernatant of lung tissue homogenates (100 µL) was mixed with 360 µL of sodium pyrophosphate (0.1 M, pH 8.3), then nicotinamide adenine dinucleotide (NAD) (150 µL), 150 µl nitro tetrazolium blue (NBT) 0.3 mM, and finally, 50 µl phenazine methosulfate (PMS) 0.93 mM was progressively added. The reaction started quickly after fast addition of PMS, and the change in absorbance was measured at 560 nm at time intervals of 0, 1, 2, 3, 4, and 5 min until the color change stopped. In the blank tube, 100 µL of saline was added instead of the supernatant. Using the following equation, SOD activity was calculated:

Inhibition of reduction of NBT dye = rate of change of blank – rate of change of sample/rate of change of blank ×100.

SOD activity (U/mg tissue) = SOD inhibition ratio ×3.75 × 1/g of tissue used.

#### Catalase enzyme assay

By using the procedures outlined by Biodiagnostic Group, Egypt. The supernatant from lung tissue homogenate (15 µl) was mixed with 150 µl of phosphate buffer (pH 7.0) and 30 µl of substrate (H_2_O_2_). The mixture was then incubated for one minute at 25 °C, followed by adding 60 µl of chromogen inhibitor and 150 µl of peroxidase 4-aminoantipyrine preservative (enzyme). The mixture was incubated for another 10 min at 37 °C. In the blank tube, distilled H_2_O (15 µl) was added instead of the supernatant and the other reagents were added as in sample tube. Finally, the absorbance of the sample and blank was measured at 520 nm. The hydrogen peroxide-scavenging activity of the samples was calculated as follows:

Catalase activity in tissue (U/g) = A _standard_ – A _sample_ / A _standard_ × 1/ g tissue used per test.

#### Lipid peroxidation (Malondialdehyde level assay)

Malondialdehyde (MAD) was determined according to the method of Stocks and Donnandy, with slight modifications^43^. This method is based on the reaction between thiobarbituric acid (TBA) and MDA (a lipid peroxidation product) to form a colored compound that can be measured colorimetrically. Briefly, PBS (800 µl) was added to 200 µL of lung tissue homogenate, then 500 µL of 30% trichloroacetic acid (TCA) was added, then mixed well and placed on ice for at least 2 h, then centrifuged at 2000 rpm for 20 min. The supernatant (1000 µl) was aspirated from each sample separately and transferred to another tube. Then 75 µl of EDTA (0.1 M) and 250 µl of TBA (1%) in NaOH were added to each tube. They were then mixed well and placed in a boiling water bath for 10 min, then immediately cooled until they reached room temperature. The absorbance of each tube is measured and recorded at 532 nm and 600 nm. The MDA activity of the samples was calculated as follows:

MDA (mole/g tissue) = (A_532_ – A_600_) × 7.5 × 1.325 × 100/1.56 × 10^5^ × Total protein.

Where, 1.56 × 10^5^ = the molar extinction coefficient of the MDA-TBA complex at 532 nm, 7.5 = dilution during extraction, and 1.325 = dilution during color development.

#### Flow cytometry study of CD44

Lung tissues of different groups of mice were first dissociated into a single cell, then suspended in phosphate-buffered saline with 1% BSA, and the cell concentration was adjusted using PBS/BSA buffer to one million cells/ml. Next, three test tubes were filled with 100 µL of the cell suspension, and 10 µL of anti-CD44 antibody was added. Following thorough mixing the samples were incubated for 30 min at room temperature. The fluorochrome is directly bonded to the primary antibody throughout the incubation period. Following incubation, the cells were centrifuged at 1200 rpm for 5 min, followed by a wash with 2 mL of PBS/BSA. The supernatant was then discarded. The BD Accuri C6 Flow Cytometer from BD Biosciences was used for analysis after re-suspension of the cell pellet in 0.2 mL of 0.5% paraformaldehyde in PBS/BSA.

#### Immunohistochemistry determination of NF-κB protein

NF-κB was detected in the lung tissues of different groups of mice by PolyQ stain 2 step detection system goat anti-mouse/rabbit HRP, peroxidase quench, DAB kit. Tumor tissues were left overnight at room temperature in 10% formalin solution and then dehydrated for protection by cooling in fresh 20–30% sucrose for 16–48 h at 4 °C. Tumor slices are then cut to a thickness of 4 μm, embedded in paraffin, and placed on glass slides before staining. Next, the slides were immersed in fresh xylene for 10 min to remove paraffin, followed by a graded series of ethanol washes, immersed in citrate buffer (pH 6.0), microwaved for 10 min and then cooled to facilitate antigen retrieval. Sections were then incubated with 3% H_2_O_2_ in PBS for 10 min at room temperature in order to deactivate endogenous peroxidase activity. Next, to avoid non-specific binding, sections were incubated with PBS containing 1% bovine serum albumin for 10 min at room temperature. Following washing in PBS, the tissue sections were incubated at room temperature for 1 h with NF-κB antibody. Then some drops of hematoxylin and eosin were applied, then the slides were gently rinsed with distilled water. The slides were examined by an image bright field microscope. The NF-κB protein was quantified by the number of positive cells/totals calculated and compared with the positive control group and control group.

#### Polymerase chain reaction for detecting FAK and MAPK

RNA extraction from mice lungs was done by Direct-zol RNA Miniprep Plus (Cat# R2072, ZYMO RESEARCH CORP. USA) and then the quality of RNA was assessed by Beckman dual spectrophotometer (USA). SuperScript IV One-Step PCR kit (Cat# 12594100, Thermo Fisher Scientific, Waltham, MA USA) was utilized for reverse transcription of extracted 2 µg RNA to produce cDNA followed by PCR in one step. The primer 3 web-based tool designed specific primers for the focal adhesion kinase (FAK) and mitogen-activated protein kinases (MAPKs) genes, as well as the housekeeping gene, GAPDH. The primers were designed by Primer 5.0 software and their sequences were as follow: FAK F: 5′ CCC AGA AAG AAG GTG AAC G 3′ and FAK R: 5′ GGTCGAGGGCATGGTGTA 3′ and MAPKs F:5′ CACAGCACCTCAGCAATGAT 3′ and MAPKs R : 5′ AGGCCTATCTTCCCAGGAAA 3′ The housekeeping gene GAPDH (with primer sequences; F: 5′ ATGGTGAAGGTCGGTGTGAACG 3′ and R: 5′ TGGTGAAGACGCCAGTAGACTC 3′ was used as a reference to calculate fold change in target gene expression. PCR results were evaluated using electrophoresis on a 2% agarose gel (ethidium bromide-stained) and imaged using UV light on a Kodak EDAS 290 Imaging System^30^.

### Statistical analysis

The results were shown as the mean ± standard error of the mean (SEM) after at least three repetitions. Minitab 18 software was used for statistical analysis. The analysis of variance (one-way ANOVA) and Tukey’s test were used to compare groups. Results were deemed significant when the P value was less than 0.05.

## Results

### Characterization of nanoparticles

#### Particle size, zeta potential, and morphological characterization of NPs

**Fig. 1:**
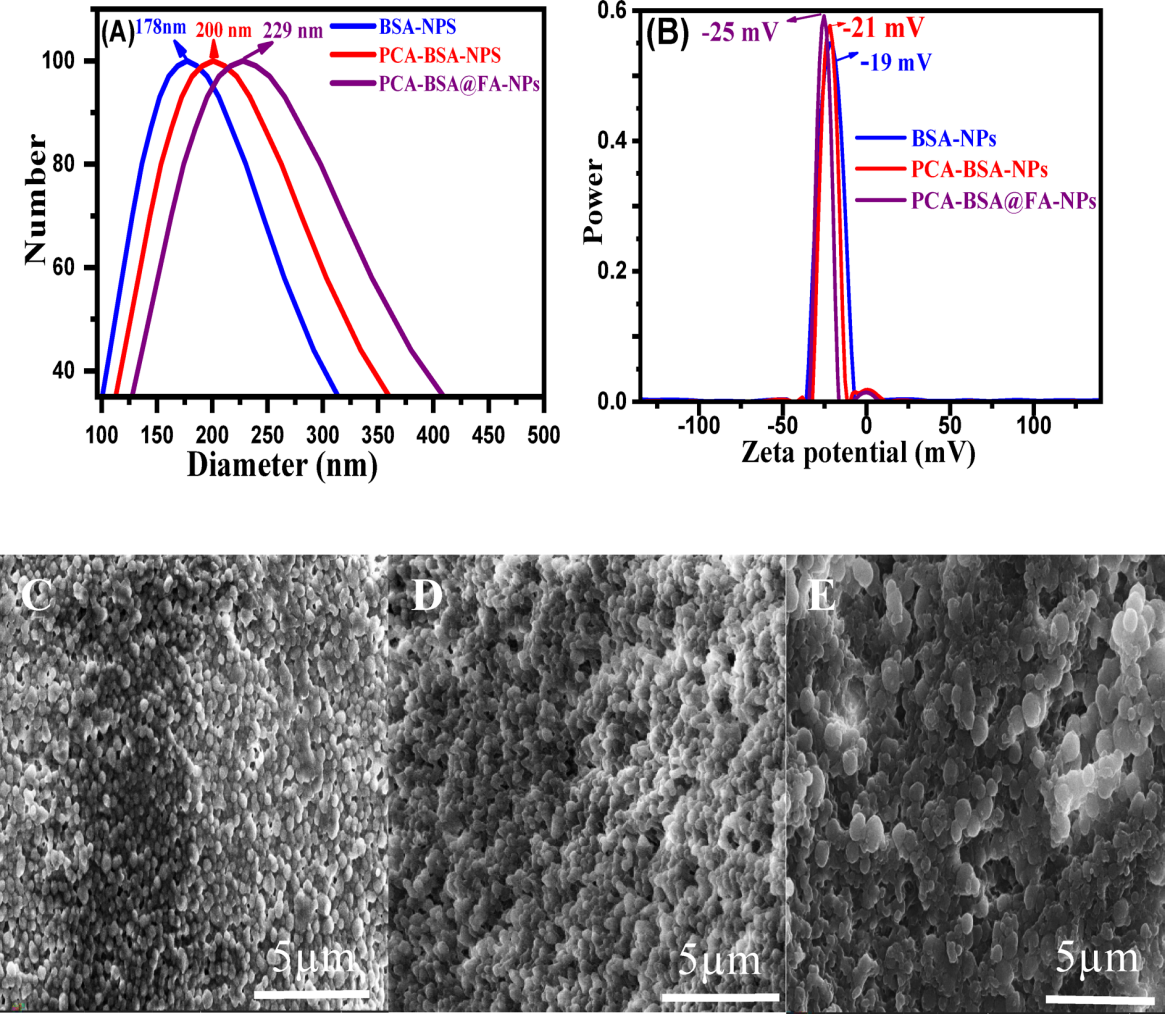
(**A**) size distribution; (**B**) zeta potential of BSA-NPs, PCA-BSA-NPs, and PCA-BSA@FA-NPs; (**C**) FESEM image of the BSA-NPs; (**D**) PCA-BSA-NPs; and (**E**) PCA-BSA@FA-NPs.

BSA-NPs, PCA-BSA-NPs, and PCA-BSA@FA-NPs had average particle sizes of 178 nm, 200 nm, and 229 nm, respectively (Fig. 1A). We evaluated the uniformity of particle size distributions using PDI. The particles are homogeneous and stable if the PDI value is less than 0.7, and the PDI values of BSA-NPs, PCA-BSA-NPs, and PCA-BSA@FA-NPs were 0.184, 0.191, and 0.192, respectively, indicating a uniform distribution of the particles^31^. The zeta potentials of BSA-NPs, PCA-BSA-NPs, and PCA-BSA@FA-NPs were − 19 mV, -21 mV, and − 25 mV, respectively (Fig. 1B, C, D, and E depict the smooth spherical surfaces of BSA-NPs (Fig. 1C), PCA-BSA-NPs (Fig. 1D), and PCA-BSA@FA-NPs (Fig. 1E), respectively, illustrating the morphology. properties of nanoparticles obtained by FE-SEM. Although the overall shape does not change, the size of the nanoparticles does. Figure 1 illustrates that BSA-NPs have a rough and granular texture, indicating a densely packed structure; however, the individual particles are challenging to distinguish clearly, pointing to significant aggregation. The particles appear to form a clustered, irregular mass with minimal visible porosity and a high degree of aggregation. In contrast, when PCA is loaded onto the BSA-NPs to create PCA-BSA-NPs, the resulting particles remain granular but show increased porosity and less density compared to the original BSA-NPs. The morphology of the PCA-BSA-NPs is somewhat more distinct, displaying irregular or rounded shapes with noticeable porosity, suggesting that the incorporation of PCA has altered the particle structure. Although aggregation persists, the overall structure is less compact than that of the BSA-NPs. Moreover, the PCA-BSA@FA-NPs exhibit a smoother texture compared to both the BSA-NPs and PCA-BSA-NPs. These particles appear to be more fused or interconnected. The morphology of PCA-BSA@FA-NPs is more defined, with rounded or elliptical shapes and reduced porosity when compared to PCA-BSA-NPs. While aggregation is still evident, the particles in PCA-BSA@FA-NPs are more distinct. The SEM images indicated that particle size increased after coupling with FA. This enhancement improved the nanoparticles’ cohesion, resulting in a rounder shape and clearer boundaries between them. The agglomeration of PCA-BSA-NPs may result from the binding of PCA to BSA-NPs, which is done by hydrogen bonds, electrostatic interactions, and hydrophobic interactions (Fig. 1D). Covalent coupling occurred between the surfaces of PCA-BSA-NPs and activated FA. Therefore, it can be said that the nanoparticles’ negative charge, both before and after the FA was bonded to their carboxyl groups on the surface, gave the produced formulations good physical stability. In contrast to PCA-BSA-NPs, PCA-BSA@FA-NPs have a greater negative surface charge. This could be because folic acid conjugates with the amino groups (NH_2_) of the nanoparticles, which lowers the positive surface charge^12,13,32^.

#### Absorption spectroscopy of BSA nps, PCA-BSA-NPs, and PCA-BSA@FA-NPs

**Fig. 2:**
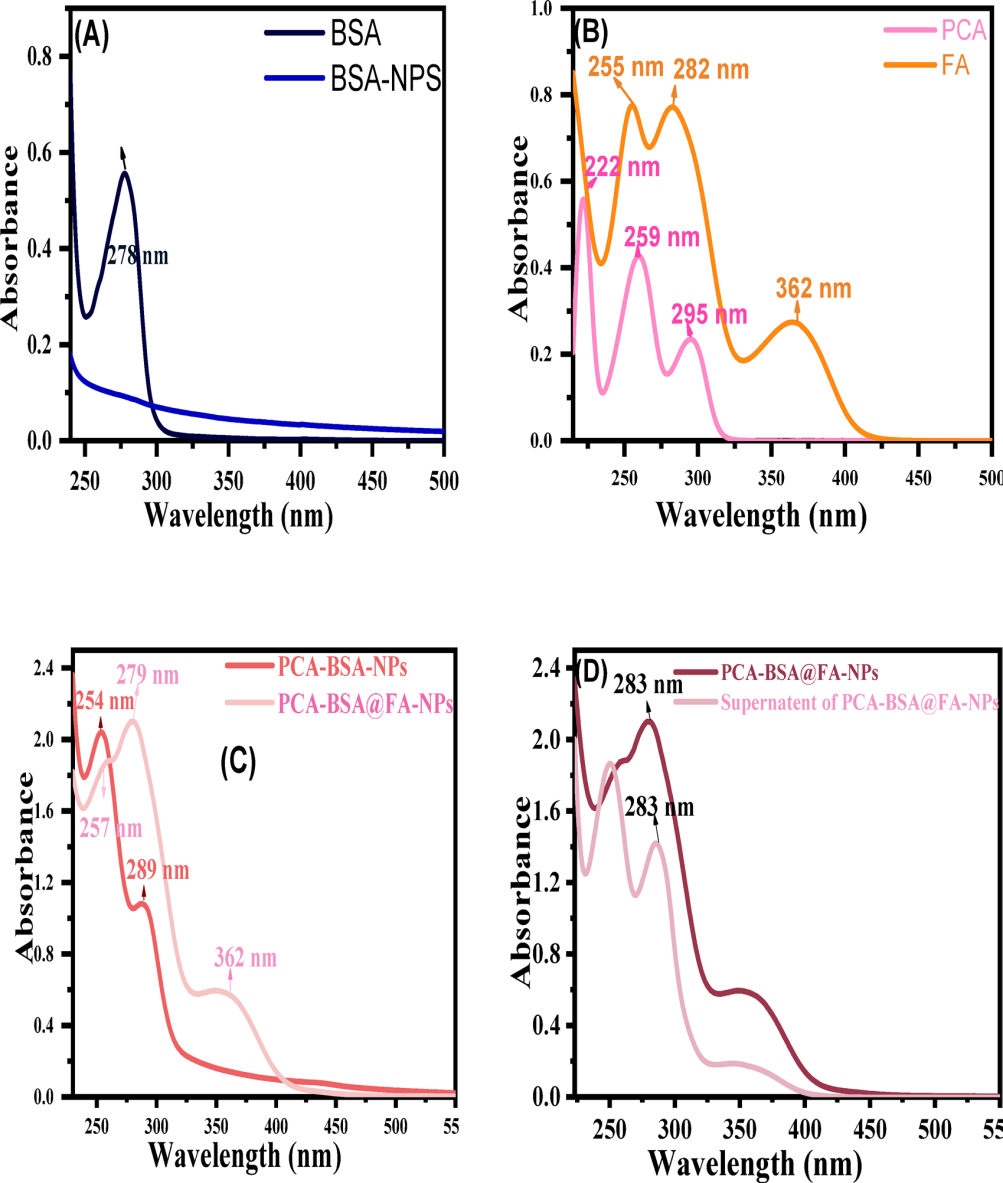
UV spectra (**A**) for BSA and BSA-NPs; (**B**) PCA and FA; (**C**) PCA-BSA-NPs and PCA-BSA@FA-NPs; and (**D**) loading capacity of PCA onto FA-BSA-NPs.

A significant difference was observed between the UV-visible spectrum of BSA, BSA-NPs, PCA, FA, PCA-BSA-NPs, and PCA-FA-BSA NPs (Fig. 2). When comparing BSA and BSA-NPs, there was a serious change in the BSA peak. This change in the BSA backbone indicates the formation of BSA-NPs. The absorption spectra of BSA and BSA NPs showed a distinct band centered at 278 nm, with no peak shift in wavelength (Fig. 2A). More interestingly, BSA-NPs showed peak broadening compared to BSA, and the peak at 278 nm is associated with weak absorption of tryptophan (Trp), phenylalanine (Phe), and tyrosine (Tyr). The data presented unambiguously suggest that certain conformational shifts are a result of NP production and might also be ascribed to the distinct conformations of BSA-NPs. The characteristic peaks for PCA adsorption are 222 nm, 259 nm, and 295 nm. In contrast, the PCA adsorption peak shifted from 259 nm to 254 nm, the 295 nm to 289 nm shift, and the 222 nm peak vanished upon encapsulating within BSA-NPs (Fig. 2B, C). FA has absorption bands at 255 nm, 282 nm, and 364 nm, which were associated with the π-π* and n-π* electronic transitions of the pterin ring and the p-amino benzoyl acid moiety, respectively (Fig. 2B). The bands of PCA-BSA-NPs are 254 nm and 289 nm. When FA is associated with PCA-BSA-NPs, some peaks disappear or the intensity decreases, and some peaks appear, indicating the association of FA with PCA-BSA-NPs. The intensity of the peak at 254 nm for PCA-BSA-NPs decreased and slightly shifted to 257 nm in PCA-BSA@FA-NPs, and the intensity of the peak at 289 nm for PCA-BSA-NPs increased and slightly shifted to 279 nm in PCA-BSA@FA-NPs (Fig. 2C). Using the PCA standard curve, the amount of PCA loaded on PCA-BSA@FA-NPs was calculated, and it was discovered that PCA-BSA-NPs had a loading effectiveness of roughly 67.2% (Fig. 2D). By comparing the percentage of PCA that was successfully contained within the nanoparticles (PCA-BSA@FA-NPs) to the total amount initially employed, the entrapment efficiency of PCA was determined to be 76% (Fig. 2B,D). Folic acid was conjugated with PCA-BSA-NPs by 33% (Fig. 2B,C). The yield value of PCA-BSA@FA-NPs equals 60%.

#### Spectral characterization

**Fig. 3:**
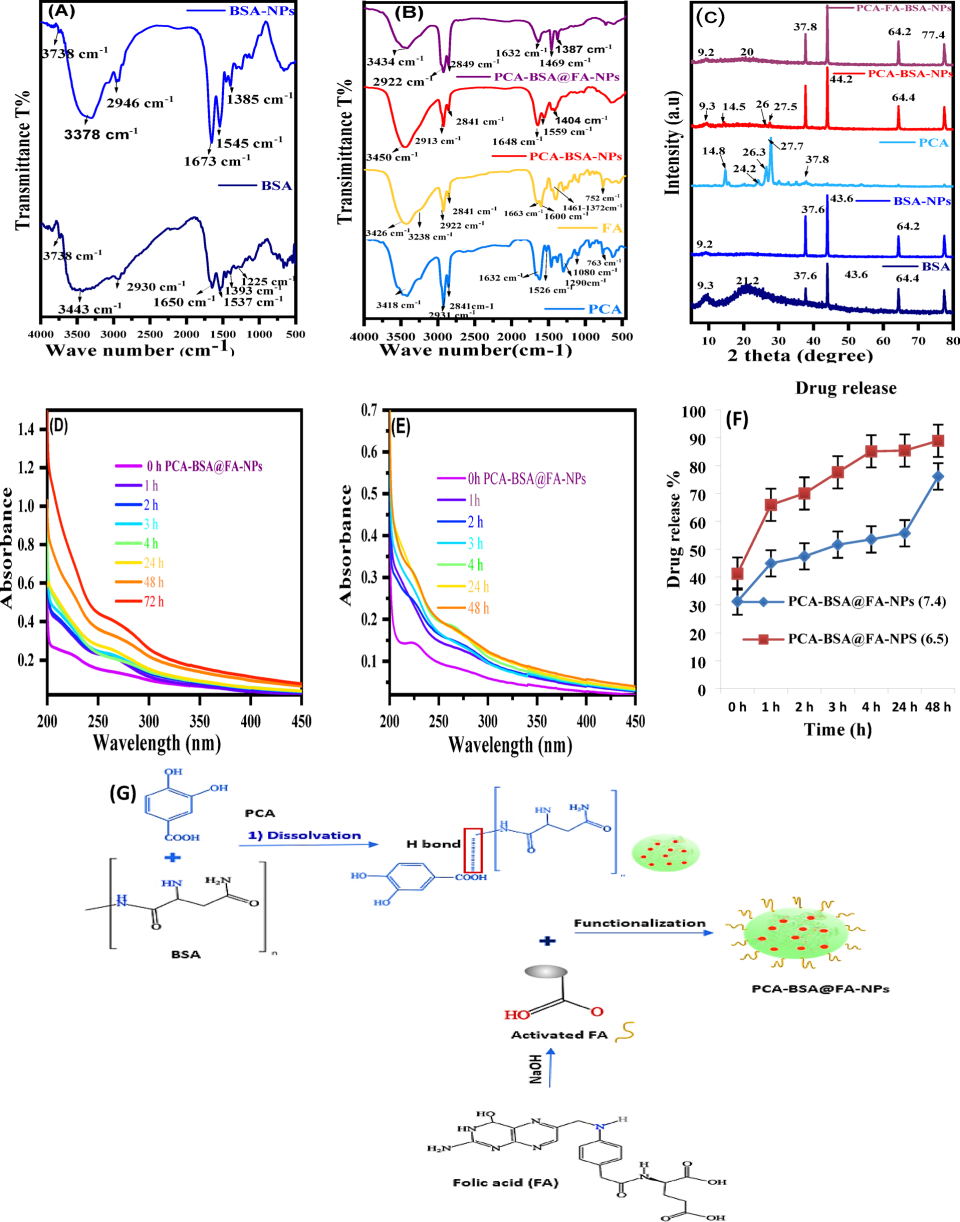
(**A**) FTIR spectra of BSA and BSA-NPs; (**B**) FTIR spectra of PCA, FA, PCA-BSA-NPs, and PCA-BSA@FA-NPs; (**C**) X-ray diffraction peaks of BSA, BSA-NPs, PCA, PCA-BSA-NPs, and PCA-BSA@FA-NPs; (**D**) UV visible absorbance of in vitro release of PCA from PCA-BSA@FA-NPs at PH 7.4; (**E**) at PH 6.5; (**F**) drug release profile of PCA from PCA-BSA-NPs; and (**G**) schematic illustration of PCA-BSA@FA-NPs preparation.

FTIR spectrum analysis of BSA and BSA-NPs confirms the formation of BSA-NPs where BSA contains major bands at 3443 cm^− 1^ (amide A, bonded by N-H stretch), 2930 cm^− 1^ (amide B, N-H stretch NH_3_^+^ free ion), 1650 cm^− 1^ (amide I, C = O stretching), 1537 cm-1 (amide II, related to C-N stretching and N-H bending vibrations), 1393 cm^− 1^ (CH_2_ bending groups), and ~ 1225 cm^− 1^ (amide III, related to C-N stretching and N-H bending). When BSA-NPs were formed, some changes occurred in the amides I (NH_2_) (1673 cm^− 1^), II (NH_3_) (1545 cm^− 1^), and III (1385 cm^− 1^) which showed a noticeable small shift at 3378 cm^− 1^, which was broader and stronger, indicating the successful formation of Nano albumin (Fig. 3A). FTIR analysis of PCA revealed its main bands, namely a hydroxyl band (3418 cm^− 1^), aromatic C-H bands (2931 cm^− 1^), a carbonyl (C = O) band (1632 cm^− 1^, aromatic (C = C) bands at 1526 cm^− 1^, and the group of bands between 1500 and 1400 cm^− 1^, the C-O band at 1290 cm^− 1^ and the C-H bending band at 763 cm^− 1^ (Fig. 3B). When PCA is encapsulated within BSA-NPs, some bands disappear or decrease in intensity, while others increase in intensity and clarity when compared to the bands related to PCA and BSA-NPs. For example, changes in the amides I (NH_2_) (1648 cm^− 1^), II (NH_3_) (1559 cm^− 1^), and III (1404 cm^− 1^) show a noticeable small shift at 3450 cm^− 1^, which is broader and clearly strong. This indicates that the chemical reactions of these groups took place through hydrogen bonding supporting the PCA-encapsulated NP (Fig. 3B). Additionally, a significant decrease in intensity was observed in the amide B and amide III bands, indicating alterations in the C-N and/or NH bonds as a result of interactions between various groups on BSA (Fig. 3A). This indicates that some bands displayed intensity differences. FTIR analysis of FA showed the hydroxyl (OH) stretching bands of the glutamic acid moiety and the NH– group of the pterin ring are responsible for the bands for FA between 3500 and 3200 cm^− 1^. At 1663 cm^− 1^, the stretching vibration peak of C = O is seen, while the bending mode of NH– vibration is associated with the band at 1600 cm^− 1^. The bands ranging from 1461 cm^− 1^ to 1372 cm^− 1^ are identified as the phenyl and pterin ring’s distinctive absorption band. Following the conjugation of folic acid with PCA-BSA-NPs, the C = O band was moved to a lower wavelength (1632 cm ^− 1^), indicating the creation of an amide bond between the amine group of PCA-BSA-NPs and the carboxylic acid group of folic acid (Fig. 3B)^13,17^. First, folic acid was activated using NaOH. Then preactivated FA solution was added slowly to the stirring PCA-BSA-NP solution. Activated FA was coupled covalently with the surface of PCA-BSA-NPs. This reaction was kept overnight under continuous stirring at room temperature (Fig. 3G)^13,33^.

#### X-ray diffraction

The reflections of BSA, BSA-NPs, PCA, PCA-BSA-NPs, and PCA-BSA@FA-NPs in XRD are shown in Fig. 3C. The evolution of BSA-NPs is indicated by comparing it with BSA diffraction, where BSA displays sharp peaks at 2θ = 9.3°, 21.2°, 37.6°, 43.6°, 64.2°, and 77.6°, while BSA-NPs show the same peaks of BSA with different intensities and the disappearance of the peak at 21.2°. The broad peaks of BSA are associated with the small particle size, which agrees with the SEM results. The PCA showed sharp reflections at 2θ = 14.8º, 27.7°, and 26.3º, as well as a small peak around 24.2° and 37.8°, which correspond to the PCA inversion. After the formation of PCA-BSA-NPs, the intensity of the two peaks at 27.5º and 14.4º was diminished. Some peaks of BSA-NPs and PCA showed intensity fluctuations, indicating the generation of PCA-BSA-NPs and a rise in the crystallinity of the nanocomposite. In previous studies, X-ray of FA was shown as it possesses sharp peaks at angles of 2θ = 10º, 12.6º, 15º, 21º,26º, 28.9º, as well as a small peak around 32º, 34º, and 36º^33^. When FA is conjugated with PCA-BSA-NPs few peaks are shifted Whether in PCA-BSA-NPs or FA. Peak at 14.5º, 26º, and 27.5º in PCA-BSA-NPs disappear. The disappearance of some peaks and the difference in intensity of others indicate the binding of FA to the nanocomposite.

#### ^1^H NMR of BSA-NPs, PCA-BSA-NPs, and PCA-BSA@FA-NPs

**Fig. 4:**
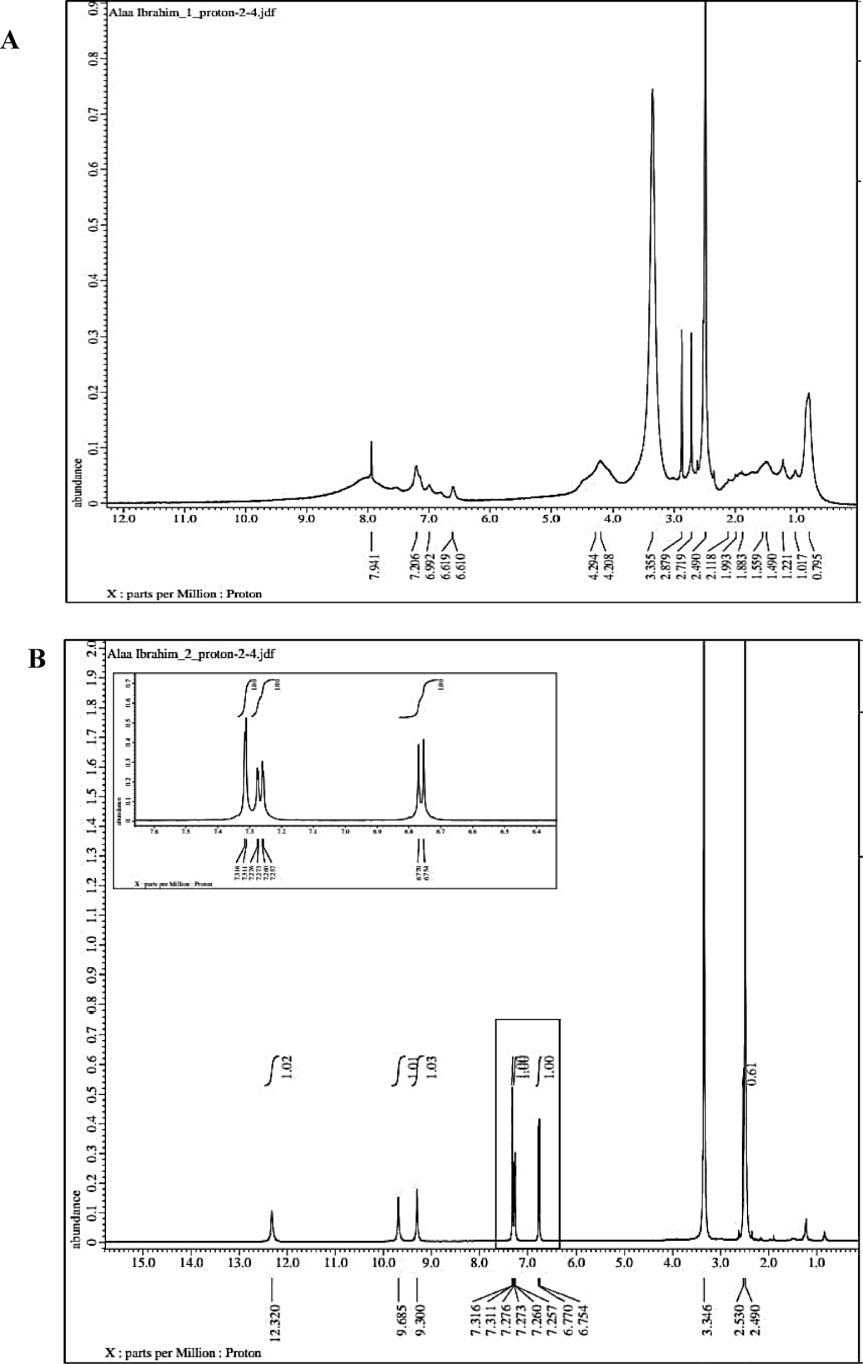

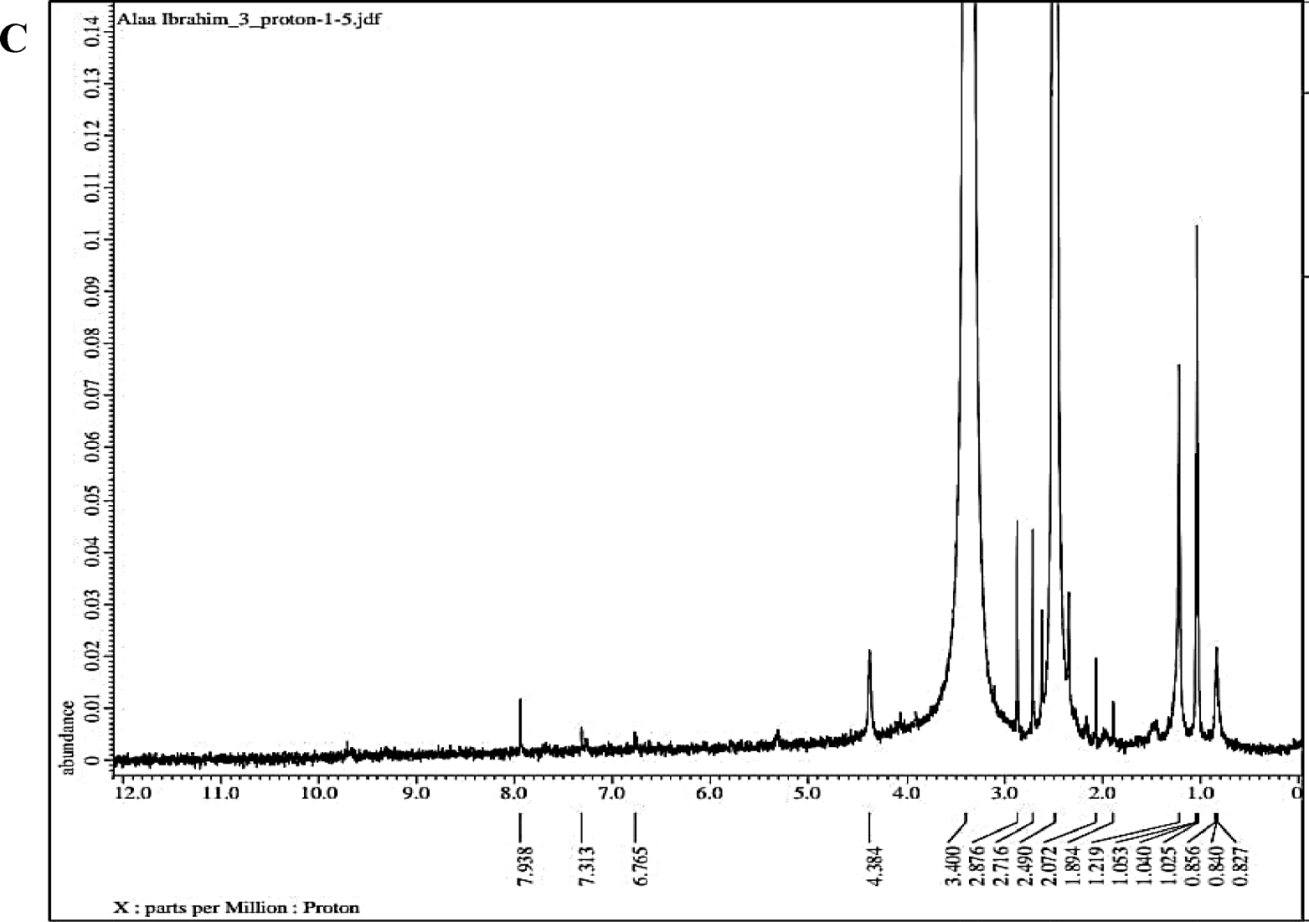
^1^H NMR of (**A**) BSA-NPs; (**B**) PCA-BSA-NPs; and (**C**) PCA-BSA@FA-NPs.

Figure 4A presents the^1^H NMR spectrum of BSA nanoparticles. In this spectrum, the chemical shifts between 7.9 and 6.6 ppm correspond to the aromatic protons of phenylalanine, tyrosine, and tryptophan residues in BSA. The region from 4.3 to 4.2 ppm is attributed to the alpha protons (CH) adjacent to the carboxylic acid groups (COOH) in the amino acids, representing the backbone signals of the protein. The shifts between 1.8 and 0.8 ppm indicate the presence of aliphatic protons (methyl and methylene groups) from leucine, isoleucine, valine, and lipid residues. The peak at 0.795 ppm reflects the terminal methyl groups at the aliphatic ends of the amino acid side chains. The well-defined peaks suggest that some secondary structure has been retained, although there is line broadening due to the formation of nanoparticles. This peak broadening, particularly in the 1.0 to 4.0 ppm region, is expected as the mobility of protons is restricted in nanoparticle form. The reduced resolution compared to free BSA in solution may indicate aggregation or crosslinking, which are common during nanoparticle synthesis. Overall, the chemical shifts are similar to those observed in native BSA, indicating that there have been no significant chemical modifications to the functional groups^34^. The^1^H NMR spectrum confirms BSA’s protein-related proton signals, indicating its intact nanoparticle form, possibly due to structural constraints or aggregation effects causing peak broadening. The^1^H NMR of BSA-NPs was displayed in Fig. 4A, where δH 7.9 to 6.6 ppm was associated with the aromatic protons of the BSA aromatic residues phenylalanine, tyrosine, and tryptophan. The backbone signals of amino acids in proteins are δH 4.3 to 4.2 ppm α-protons, which are the CH atoms adjacent to COOH. Leucine, isoleucine, valine, and fatty residues contribute 1.8 to 0.8 ppm of aliphatic protons (methyl, methylene). The aliphatic terminals of amino acid side chains have terminal methyl groups with a δH of 0.795 ppm. The study reveals well-defined peaks indicating partial retention of secondary structure, although line broadening may occur due to nanoparticle formation. The lower clarity compared to free BSA in solution might suggest that particles are sticking together or linking up, which is common when making nanoparticles. The chemical shifts resemble those of native BSA, suggesting no major chemical modification of functional groups^34^. PCA’s^1^H-NMR spectrum revealed three olefin methine proton signals: δH 7.46 (1 H, H-2), δH 7.44 (1 H, dd, J = 8.0, 2.0 Hz, H-6), and δH 6.81 (1 H, d, J = 8.0 Hz, In H-5). It was projected that a 1,3,4 trisubstituted benzene ring would exist as a result^35^. When PCA was loaded onto BSA-NPs, the aromatic region (6.5–7.5 ppm) had more intense and better-defined peaks, indicating successful non-covalent loading of PCA (hydrogen bonding) (Fig. 4B). The δH 9.3–9.7 ppm new signals are probably the hydroxyl protons of the phenolic groups in PCA, and the δH 12.3 ppm peak indicates acidic -OH groups that PCA contains, a COOH group that is engaged in hydrogen bonding. After PCA loading, there is little change to the BSA backbone; broadening is still visible but more pronounced in the aromatic area, indicating that PCA and the protein surface interact rather than aggregating randomly. Since there are no significant changes to BSA’s backbone peaks, PCA most likely interacts by hydrogen bonding or surface adsorption. The spectrum verifies that the nanoparticle matrix contains an intact PCA molecule (Fig. 4B). When PCA-BSA-NPs were conjugated with FA, the δH 6.7–7.9 ppm indicative of the aromatic region was still present, indicating the aromatic rings of PCA and the pteridine and phenyl rings of folic acid (Fig. 4C). The presence of FA was supported by new signals that arose. The presence and unaltered δH 4.3 ppm α-protons of CH next to the COOH/NH₂ portion of BSA indicate that the protein backbone is intact. Furthermore, both the BSA and FA alkyl domains contribute 1.8–0.8 ppm of aliphatic protons. The new or intensified peaks near 6.7–8 ppm suggest the presence of FA aromatic rings, including pteridine moieties and p-aminobenzoic acid (Fig. 4C). Protons next to nitrogen or oxygen of CH₂–NH–C = O are among the shifted peaks in the 2.0–4.0 ppm band that show the development of an amide bond (between FA’s carboxyl and BSA’s amine groups). This implies effective chemical conjugation rather than only surface adsorption^36,37^.

### *In vitro *drug release

Figure 3D-F shows the optimal release of PCA from protein nanoparticles in physiological and cancer environments. This release was observed by fluorescent detection of suspensions of PCA-BSA@FA-NPs in PBS. As a result of the in vitro investigation, PCA is released from PCA-BSA@FA-NPs at different times throughout the incubation period, and this release depends on the amount of encapsulated PCA and the strength of the association between PCA and BSA. According to Fig. 3F, the PCA release at pH 7.4 was 31.2%, 44.9%, 47.4%, 51.6%, 53.5%, 55.7%, and 76.1%, while at pH 6.5, it was 41.2%, 65.9%, 70%, 77.6%, 85.1%, 85.4%, and 88.9%. These results showed that the release of PCA from the nano-formulation (PCA-BSA@FA-NPs) was significantly higher in PBS at pH 6.5 compared to PBS at pH 7.4. Bovine serum albumin (BSA) is pH-sensitive, and its degradation may be accelerated in a mildly acidic environment (pH 6.5). Acidic conditions may also promote hydrolysis or destabilize the nanoparticle structure, accelerating PCA release. At pH 6.5, protonation of the functional groups on BSA and FA may weaken their binding and facilitate faster PCA release. BSA-based nanoparticles may exhibit pH-sensitive swelling behavior. At pH 6.5, increased hydration and swelling may lead to increased porosity, allowing PCA to diffuse more rapidly. A change in release kinetics at pH 6.5 compared to pH 7.4 may indicate increased matrix degradation at lower pH^38^. PCA was adsorbed on the surface of BSA-NPS, and then the active carboxylic acid in folic acid was conjugated with BSA-NPS through an amide bond to form a stable structure targeting cancer cells. Folic acid (FA) plays a crucial role in the targeted penetration of cancer cells, primarily through its interaction with the folate receptor (FR), which is overexpressed on the surface of many cancer cells^19^. Meanwhile, our findings revealed the potential release of PCA encapsulated within the FA-BSA-NPs fragments after their cellular uptake. Moreover, the PCA-FA-BSA-NPs release PCA more effectively in the cancer environment than in the physiological environment. This supports our hypothesis that the PCA-FA-BSA-NPs are biologically effective and target cancer cells than normal cells.

### In vitro cytotoxic activity

**Fig. 5:**
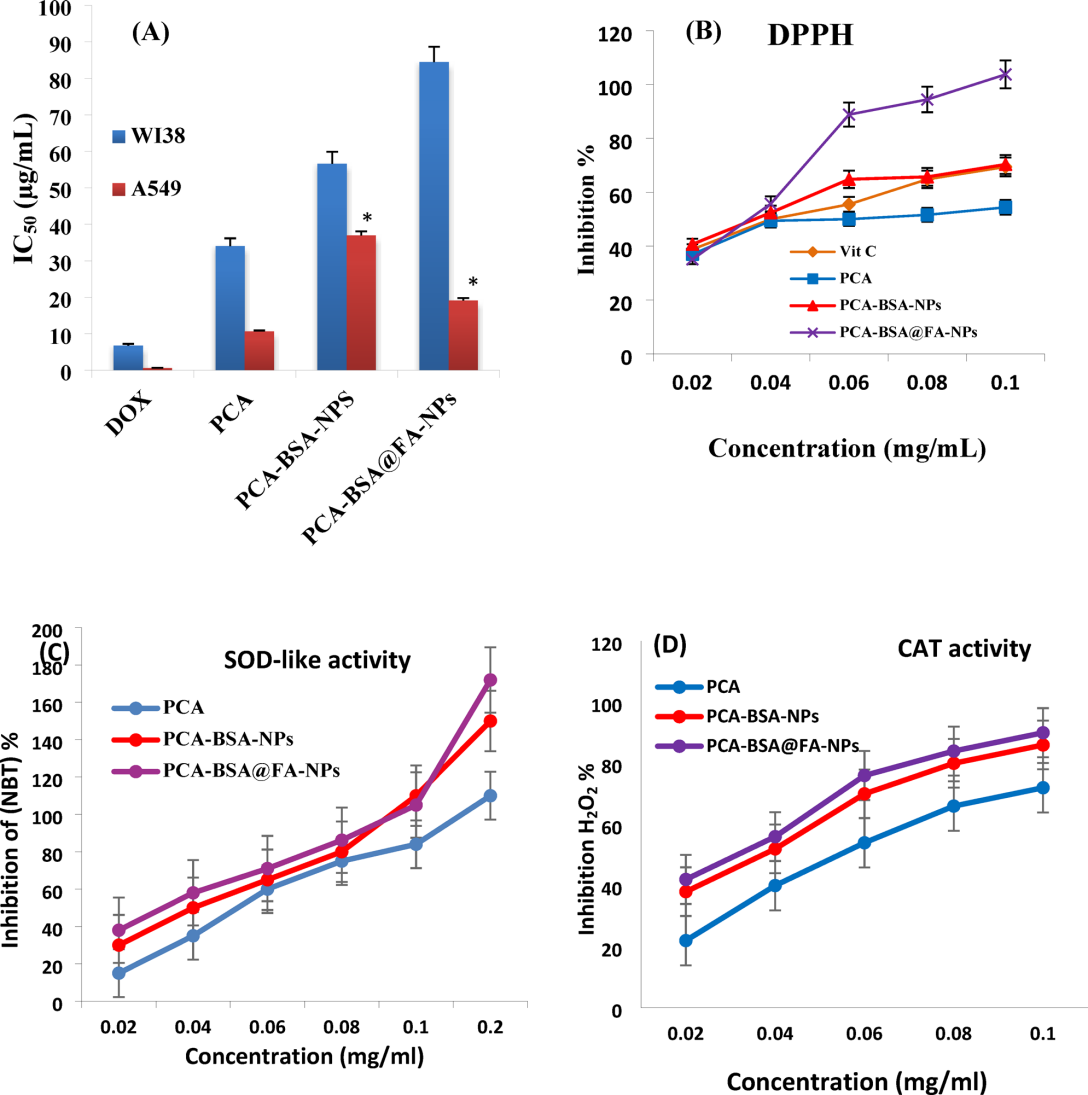
(**A**) Bar plot of the *in vitro* cytotoxicity calculations (IC_50_s) of DOX, PCA, PCA-BSA-NPs, and PCA-BSA@FA-NPs against A549 and WI38. All experiments were tested in triplicate; (**B**) The DPPH % inhibition curve by PCA, PCA-BSA-NPs, and PCA-BSA@FA-NPs. * *p <* 0.05 significant compared with the cytotoxicity of PCA, (**C**) Percentage reduction of NBT inhibition with increasing the concentration of PCA, PCA-BSA-NPs, and PCA-BSA@FA-NPs; (**D**) Percentage inhibition of H_2_O_2_ with increasing the concentration of PCA, PCA-BSA-NPs, and PCA-BSA@FA-NPs.

A549 and WI38 were used to evaluate the cytotoxicity of PCA, PCA-BSA-NPs, and PCA-BSA@FA-NPs compared with doxorubicin (a standard anticancer drug). The IC_50_ indicates the concentration at which cancer cells lose 50% of their viability. Our results showed that by increasing concentrations of PCA, PCA-BA-NPs, and PCA-BSA@FA-NPs, the inhibition of A549 cells increased. Figure 5A showed that the IC_50_ value of PCA against A549 cells (10.62 ± 0.26 µg/mL) was significantly lower than those of PCA-BSA-NPs (36.9 ± 1.14 µg/mL) and PCA-BSA@FA-NPs (19.1 ± 0.68 µg/mL) (*p =* 0.0001 for all). Our data showed that PCA-BSA@FA-NPs had stronger antiproliferative activity than PCA-BSA-NPs, with the IC_50_ value against A549 being significantly lower (1.93-fold) than that of PCA-BSA-NPs (*p =* 0.0001). However, compared to doxorubicin (0.58 ± 0.08 µg/mL), the IC_50_ of PCA, PCA-BSA-NPs, and PCA-BSA@FA-NPs against A549 cells were significantly increased (*p =* 0.0001 for all). Additionally, PCA-BSA@FA-NPs exhibited significantly lower toxicity compared to DOX (12.56-fold), PCA (2.48-fold), and PCA-BSA-NPs (1.49-fold), as indicated by their IC_50_ values against WI38 normal cells, which were considerably higher than those of DOX, PCA, and PCA-BSA-NPs (*p =* 0.0001 for all). Although PCA is effective against A549 cells, it has a toxic effect on WI38 normal cells (IC_50_ = 34.04 ± 0.26 µg/mL). As a result, our data show that PCA-BSA@FA-NPs are more effective in selectively targeting cancer cells than PCA. Therefore, the PCA encapsulated within BSA-NPs and its conjugation with FA increased its selectivity for cancer cells without harming normal cells. The purpose of conjugating folic acid (FA) is based on our hypothesis that it significantly increases the expression of the folate receptor (FR) in various human cancer cells, including those in the lungs. In contrast, the expression of this receptor is low in healthy tissues^39,40^. Folic acid has a unique ability to bind to folate receptors. Additionally, FA is non-immunogenic and stable across a wide range of pH levels, making it suitable for use in the cancer environment^41^. Folates enter cells via endocytosis^42^attaching to receptors that may facilitate the entry of PCA-BSA@FA-NPs into cancer cells.

### DPPH scavenging activity

The ability of PCA, PCA-BSA-NPs, and PCA-BSA@FA-NPs to neutralize the free radicals was determined through their DPPH scavenging activity, demonstrated by their ability to decolorize DPPH solution^43^. In our data, non-significant change in DPPH IC_50_ values was observed in PCA-BSA-NPs (*p =* 0.400) and PCA-BSA@FA-NPs (*p* = 0.125) compared to standard vitamin C (1.78 ± 0.408, 1.55 ± 0.408, and 2.250 ± 0.408 mg/mL, respectively) (Fig. 5B).Moreover, there were significant increases in the DPPH IC_50_ values of PCA-BSA-NPs (*p =* 0.001) and PCA-BSA@FA-NPs (*p* = 0.0001) compared to PCA (3.250 ± 0.250 mg/mL), which indicates their improved ability to scavenge the ROS. As a result, PCA-BSA-NPs and PCA-BSA@FA-NPs showed high antioxidant activity that was almost as strong as vitamin C and stronger than PCA. Therefore, PCA-BSA-NPs and PCA-BSA@FA-NPs demonstrated potent antioxidant activity that was stronger than PCA and nearly as powerful as vitamin C.

### SOD-like activity

The SOD-like activity assay is based on the inhibition of NBT by PCA, PCA-BSA-NPs, or PCA-BSA@FA-NPs. The principle of this test is based on the ability of PCA, PCA-BSA-NPs, and PCA-BSA@FA-NPs to transfer electrons or O_2_^•−^ to react with NBT, converting the yellow tetrazolium to a blue precipitate^44^. In our data, a significantly decreased IC_50_ value for SOD-like activities was detected for PCA-BSA-NPs (*p =* 0.024 ) and PCA-BSA@FA-NPs (*p =* 0.005 ) compared to PCA (2.81 ± 0.36, 1.55 ± 0.27, and 1.90 ± 0.25 mg/ml, respectively). Non-significant change in IC_50_ value for SOD-like activities was detected for PCA-BSA-NPs compared to PCA-BSA@FA-NPs (*p =* 0.37). This indicates the ability of PCA-BSA-NPs and PCA-BSA@FA-NPs to remove ROS and higher antioxidant activity compared to PCA (Fig. 5C).

### Catalytic activity assay

The catalytic activity of PCA, PCA-BSA-NPs, and PCA-BSA@FA-NPs was assessed by their ability to dissociate H_2_O_2_ and liberate O_2_. Our experiments showed that PCA-BSA-NPs have higher catalytic activity than PCA (Fig. 5D). Thus, the IC_50_ values for PCA, PCA-BSA-NPs and PCA-BSA@FA-NPs were 3.03 ± 0.55, 2.07 ± 0.36, and 1.17 ± 0.20 mg/ml, respectively. PCA-BSA-NPs and PCA-BSA@FA-NPs had a significantly lower IC_50_ value than PCA (*p* = 0.026 and 0.001, respectively), indicating that they are more effective in degrading H_2_O_2_ (Fig. 5D). A significant decrease in IC_50_ value was noticed in PCA-BSA@FA-NPs compared to PCA-BSA-NPs (*p =* 0.034). This data, along with the previous DPPH scavenging study and SOD-like activity, demonstrates the higher antioxidant capability of the PCA-BSA-NPs when compared to PCA alone. Previous study established that PCA has antioxidant activity by scavenging ROS^5^. That result agreed with our hypothesis that encapsulating PCA within the BSA-NPs would increase their antioxidant capacity. Furthermore, the increased antioxidant capacity of PCA-BSA@FA-NPs compared to PCA-BSA-NPs was because of conjugated FA. As a result, the PCA-BSA@FA-NPs will be used in animal experiments.

### Experimental animals’ results

#### LD_50_ result

After oral administration of PCA-BSA@FA-NPs and observing no death or sign of toxicity in the mice groups, the LD_50_ value was observed to be above 2000 mg/kg. In previous research, the permissible oral dose of PCA in mice was 50 mg/kg for seven consecutive days dissolved in corn oil^45^. A high dose of PCA-BSA@FA-NPs, specifically 50 mg/kg, dissolved in distilled water, was administered similarly to the dose used for PCA. In addition, half the dose of PCA-BSA@FA-NPs, representing 25 mg/kg dissolved in distilled water (the low dose), was used.

#### Liver and kidney function parameters

Our results showed no significant change in liver function tests (ALT and AST) levels between study groups (Table 1). There was a significant increase in albumin levels in the PCA-control group compared to the positive control group (*p =* 0.038). Also, there was a significant increase in the albumin levels in the low-dose PCA-BSA@FA-NPs-control group compared to the PCA-control group (*p* = 0.018). There was no significant change in creatinine levels in all treated groups compared to positive control, healthy control, and PCA-treated groups (Table 1). A significant increase in creatinine levels was noticed in the PCA-control group compared to the healthy control group (*p =* 0.0001), indicating the toxic effect of PCA on the kidneys. Also, significant decreases in creatinine levels were observed in all treated groups compared to the PCA-control group (*p =* 0.0001, for all). These data indicated that the PCA-BSA@FA-NPs nano-formulation has reduced the toxicity of PCA, rendering it safe for the kidneys.

**Table 1:** Liver and kidney function tests in different study groups.

Groups	Albumin (g/dl)	ALT (U/L)	AST (U/L)	Creatinine (mg/dl)
Healthy control	4.225 ± 0.911 ^a^	51.25 ± 14.3 ^a^	315.0 ± 35.1 ^a^	1.355 ± 0.604 ^a^
PCA-control	4.887 ± 0.131 ^#^	34.25 ± 5.91	203.0 ± 53.9	3.938 ± 0.206 ^*^
Low-dose PCA-BSA@FA-NPs-control	3.900 ± 0.949	47 ± 14.45	187.5 ± 62.4	1.625 ± 0.759 ^a^
High-dose PCA-BSA@FA-NPs-control	3.768 ± 0.450	44.25 ± 11.9	207.0 ± 12.57	1.050 ± 0.576 ^a^
Positive control	3.250 ± 0.900	62.8 ± 29.7	283.5 ± 34.5	0.892 ± 0.117
PCA-treated	3.525 ± 0.695 ^a^	48 ± 23.4	297.0 ± 46.0	0.965 ± 0.539 ^a^
Low-dose PCA-BSA@FA-NPs-treated	3.075 ± 0.222	45.8 ± 21.3	247.5 ± 66.9	0.990 ± 0.613 ^a^
High-dose PCA-BSA@FA-NPs-treated	3.125 ± 0.802	42.0 ± 20.5	222.3 ± 104.0	1.010 ± 0.560 ^a^

Values represented by mean ± SEM. * *p <* 0.05 significant compared with the healthy control group, ^#^ *p <* 0.05 significant compared with the positive control group, and ^a^ *p* < 0.05 significant compared with the PCA-control group.

#### Bodyweight change

**Fig. 6:**
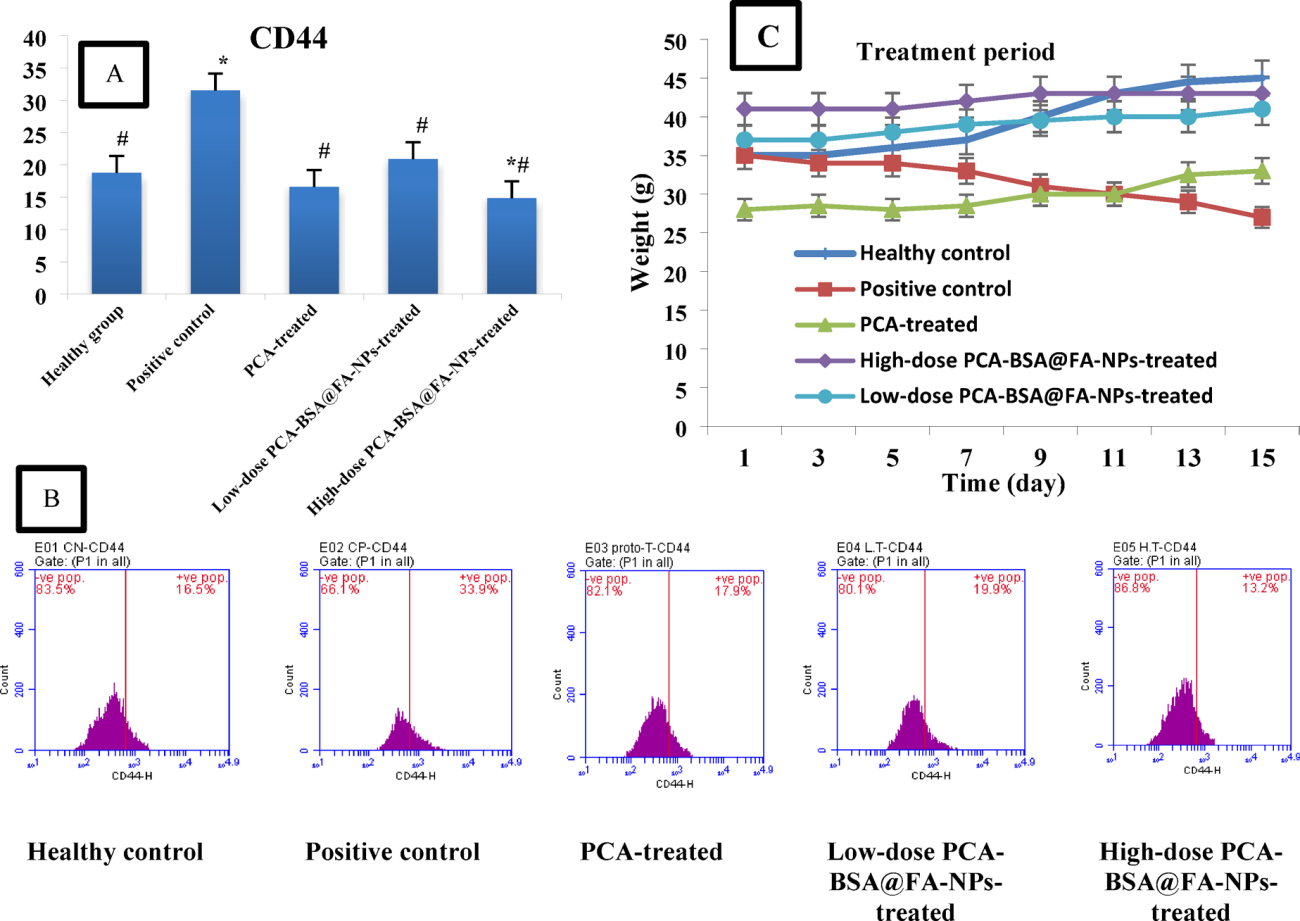
(**A**) Bar plot of the CD44 expression level represented by mean ± SEM. (**B**) Flow cytometry images of the CD44 expression in lung tissue of different groups. (**C**) Mean body weight change (g) of the study mice groups during the treatment period (15 days). * *p <* 0.05 represented significantly compared with the healthy control group, and ^#^ *p <* 0.05 represented significantly compared with the positive control group.

Our data indicated no significant changes in body weight among all control groups compared with the healthy control group. The changes in body weights during the treatment period of all treated groups compared to the positive control group are illustrated in Fig. 6C. Significant increases in body weight were observed in the high-dose PCA-BSA@FA-NPs, low-dose PCA-BSA@FA-NPs, and PCA-treated groups compared to the positive control group (*p* = 0.0001 for all). However, there were no significant differences in body weight between the low-dose and high-dose PCA-BSA@FA-NPs-treated groups compared to the healthy control group (*p* = 0.109 and *p* = 1, respectively). In contrast, the PCA-treated group showed a significant reduction in body weight compared to the healthy control group (*p* = 0.0001, Fig. 6C). These findings confirm that both low-dose and high-dose PCA-BSA@FA-NP treatments are effective in reducing the burden of lung cancer. As a result, the body weight in these treatment groups increased, reaching levels comparable to those of the healthy control group. Conversely, the weight loss seen in the PCA-treated group may be attributed to a slightly toxic effect of PCA, as indicated by increases in creatinine levels, or it may be related to the cancer burden that was not completely mitigated by the treatment.

#### Antioxidant enzymes activity assay

The effect of PCA and PCA-BSA@FA-NPs on oxidative stress markers is shown in Table 2. Reactive oxygen species (ROS) levels are significantly elevated in cancer cells, leading to DNA, tissue, and organ damage^46^. The function of antioxidant enzymes is to neutralize different forms of ROS. However, when these levels rise as a result of cancer, the activity of these enzymes, including SOD, and CAT decreases, while oxidative stress marker MDA increases^46,47^. To prevent cell damage, SOD scavenges superoxide anion, while CAT scavenges intracellular hydrogen peroxide and lessens oxidative cell damage^46^. When ROS damage the lipids in cellular membranes, they produce a reactive byproduct called malondialdehyde (MDA). MDA is frequently employed as a biomarker for cellular damage and oxidative stress^47,48^. The decrease in SOD and CAT levels, and raised MDA levels, were observed in lung cancer^46–48^. Our data indicated a significant decrease in SOD levels in the lung tissues of the positive control group (51%) compared to the healthy control group (*p =* 0.001). No significant change in SOD levels in PCA-control, low-dose PCA-BSA@FA-NPs-control, and high-dose PCA-BSA@FA-NPs-control groups compared to the healthy control group. There was a significant increase (1.78 -fold) in SOD levels in the PCA-control group compared to the positive control group (*p =* 0.005). In the treated groups, the SOD levels in the high-dose PCA-BSA@FA-NPs-treated group were significantly increased by 1.16 and 2.07-fold, respectively, compared to both the PCA-treated group and the positive control group (*p =* 0.015 and 0.0001, respectively). Moreover, the levels of CAT in lung tissue were significantly decreased by 0.67-fold in the positive control group compared with the healthy control group (*p =* 0.022). There were no significant changes in CAT levels in the lung tissues in all treated groups compared with the healthy control group. There was a significant increase in CAT levels in the PCA-control (1.5-fold, *p* = 0.008), low-dose PCA-BSA@FA-NPs-control (1.47-fold, *p* = 0.023), and high-dose PCA-BSA@FA-NPs-control (1.6-fold, *p* = 0.005) groups compared to the positive control group. In contrast, no significant improvement in CAT levels in the lung tissues of the PCA-treated group was observed compared with the CAT levels of the positive control group (*p =* 0.232). However, significant increases in CAT levels were observed in both the low-dose and high-dose PCA-BSA@FA-NPs-treated groups, showing 1.7- and 1.8-fold increases, respectively, compared to the positive control group (*p* = 0.001 and 0.0001, respectively). Additionally, the CAT levels in the low-dose PCA-BSA@FA-NPs and high-dose PCA-BSA@FA-NPs treatment groups demonstrated significant increases (1.43 and 1.45-fold, respectively) compared to the PCA-treated group (*p =* 0.011 and 0.008, respectively). In contrast, the overall levels of MDA were significantly increased (3.96-fold) in the positive control group compared to those in the healthy control group (*p =* 0.0001). Table 2showed a significant decrease in MDA levels (2.16, 1.98, and 1.76-fold, respectively) in all treated groups, including PCA, low-dose PCA-BSA@FA-NPs, and high-dose PCA-BSA@FA-NPs, compared to the positive control group (*p =* 0.0001, for all). Furthermore, there was no significant change in MDA levels in the low-dose PCA-BSA@FA-NPs-treated and high-dose PCA-BSA@FA-NPs-treated groups compared with the healthy control group. However, a significant increase of 2.16-fold in MDA levels was observed in the PCA-treated group compared to the healthy control group (*p* = 0.014). In consistency with our results, previous studies have shown that PCA can decrease the oxidative stress marker MDA and scavenge free radicals by increasing the levels of SOD, CAT^5,43^. Our data showed that treatment with high-dose PCA-BSA@FA-NPs resulted in significant increases in SOD and CAT levels, along with a decrease in MDA levels, compared to both the positive control and PCA-treated groups (Table 2). As a result, we could assume that the antioxidant capacity was enhanced by PCA-BSA@FA-NPs treatment, which may reduce ROS levels and prevent cell damage.

**Table 2:** Effect of PCA and PCA-BSA@FA-NPs on lung tissue antioxidant parameters against urethane-induced lung cancer in mice.

Groups	SOD (U/mg tissue)	CAT (U/g)	MDA (mole/g tissue)
Healthy control	3432 ± 264 ^#^	141.1 ± 21.9 ^#^	0.820 ± 0.454 ^#^
PCA-control	3151.6 ± 105.8 ^#^	149.5 ± 13.77 ^#^	0.790 ± 0.130 ^#^
Low-dose PCA-BSA@FA-NPs-control	2553 ± 231	140.7 ± 17.57 ^#^	0.944 ± 0.100 ^#, a^
High-dose PCA-BSA@FA-NPs-control	2807 ± 435	153.5 ± 15.75 ^#^	0.947 ± 0.184 ^#, a^
Positive control	1767 ± 301 ^*^	95.7 ± 8.26 ^*^	3.250 ± 0.742^*^
PCA-treated	2417 ± 639	118.5 ± 11.12	1.775 ± 0.1708 ^#^
Low-dose PCA-BSA@FA-NPs-treated	2764 ± 725	169.9 ± 54.0 ^#, a^	1.625 ± 0.1708 ^#^
High-dose PCA-BSA@FA-NPs-treated	3658 ± 552 ^#, a^	172.7 ± 34.6 ^#,a^	1.450 ± 0.311^#^

Values represented by mean ± SEM. * *p <* 0.05 significant compared with the healthy control group, ^#^ *p* < 0.05 significant compared with the positive control group, and ^a^ *p <* 0.05 significant compared with the PCA-treated group.

#### CD44 expression levels

CD44 protein is highly expressed on the surface of cancer cells and plays a crucial role in cancer progression. It contributes to processes such as metastasis, invasion, adhesion, and angiogenesis^49–51^. Increased expression of CD44 has been associated with the proliferation of human lung cancer cells and their resistance to treatment^50,51^. Figure 6B illustrates a significant increase in CD44 expression by 1.67-fold in the positive control group compared to the healthy control group (*p =* 0.0001). In our data, the CD44 expression levels were restored to their level in the healthy control group in both the PCA-treated group and the high-dose PCA-BSA@FA-NPs-treated group (*p =* 0.37 and 0.40, respectively). In the group treated with low-dose PCA-BSA@FA-NPs, CD44 expression levels significantly increased by 0.21-fold compared to the healthy control group (*p =* 0.029). Moreover, significant reductions in CD44 expression (0.52, 0.66, and 0.47-fold) were observed in the PCA-treated group, the high-dose PCA-BSA@FA-NPs-treated group, and the low-dose PCA-BSA@FA-NPs-treated group compared to the positive control group (*p =* 0.0001, for all). Figure 6A indicated that no significant change in CD44 expression was observed in the high-dose PCA-BSA@FA-NPs-treated group compared to the PCA-treated group (*p =* 0.57). The low-dose PCA-BSA@FA-NPs-treated group also showed a significant increase in CD44 expression (1.25 and 1.4-fold, respectively) compared to both the PCA-treated group and the high-dose PCA-BSA@FA-NPs-treated group (*p =* 0.01 and 0.001, respectively). Our results confirmed that treatment with a high dose of PCA-BSA@FA-NPs is more effective in restoring CD44 receptor levels to normal, which may aid in lung cancer therapy.

#### Histopathological examination

**Fig. 7:**
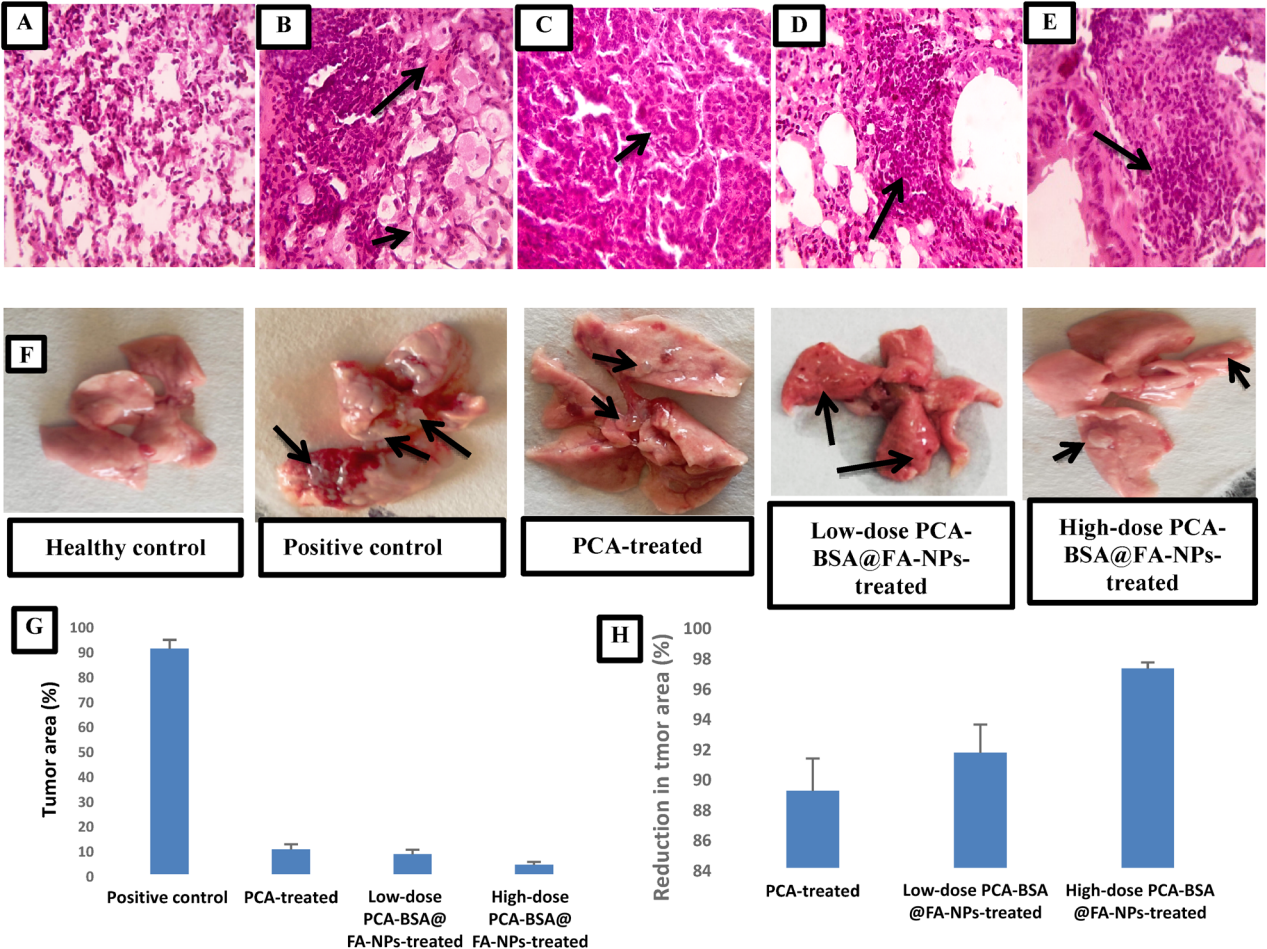
Histopathological images of lung section of (**A**) healthy control, (**B**) positive control, (**C**) PCA-treated, (**D**) low-dose PCA-BSA@FA-NPs-treated, (**E**) high-dose PCA-BSA@FA-NPs-treated groups, (**F**) illustrative images of the lung in different study groups, (**G**) Bar plot representing percentage of tumor area in studied groups, and (**H**) Bar plot representing reduction in tumor area in treated groups.

Histopathological images provide clear visual evidence of reduced tumor burden. This is evident in the images of the PCA-BSA@FA-NPs-treated groups, which showed a shrinkage in tumor area compared to the positive control groups. The PCA-BSA@FA-NPs-treated groups also showed a decrease in tumor area compared to the PCA-treated group. These images also demonstrate differences in tumor area and shrinkage between the different treatment groups, as well as between the treated groups and the positive control group. Figure 7A showed stained lung sections of the healthy control group with normal alveolar and bronchial walls and no evidence of tumor or inflammation. In the positive control group, several large bronchial adenocarcinoma nodules appeared (Fig. 7B), replacing lung tissue, which was associated with peribronchial lymphocyte accumulation and foamy macrophage accumulation filling the alveoli (thick arrows). Lung sections from the low-dose PCA-BSA@FA-NPs-treated group (Fig. 7D) exhibit fewer, smaller tumor nodules compared to the positive control group (Fig. 7B), along with an accumulation of peribronchial lymphocytes. The tumor nodules in this group are also fewer and smaller in size than those observed in the PCA-treated group (Fig. 7C), which also shows peribronchial lymphocyte accumulation (indicated by arrowheads). In contrast, lung sections from the high-dose PCA-BSA@FA-NPs-treated group (Fig. 7E) reveal a significant reduction in tumor nodules and a notable accumulation of peribronchial lymphocytes (indicated by arrowheads). Figure 7F showed that after urethane induction, all groups of mice had a number of cystic nodules. After 14 weeks, the lungs of the positive control mice were large and covered with adenocarcinoma lesions. When PCA or PCA-BSA@FA-NPs were orally administered, many cystic nodules disappeared. However, when comparing the PCA-treated group and the PCA-BSA@FA-NPs-treated group, the images showed that the PCA-BSA@FA-NPs-treated group had significantly fewer nodules. Moreover, the percentage of tumor area in the three treated groups was significantly lower than that in the positive control group (*p* = 0.0001 for all comparisons, Fig. 7G). But there was no significant difference in tumor area percentage among the three treated groups. However, the reduction in tumor area in the high-dose PCA-BSA@FA-NPs-treated group was significantly greater than in the PCA-treated group (*p* = 0.018), with no significant difference observed between the other treated groups (Fig. 7H). These data demonstrate the efficacy of high-dose PCA-BSA@FA-NPs in treating lung cancer, with improved lung tissue outcomes compared to both the PCA-treated and low-dose PCA-BSA@FA-NPs-treated groups.

#### PCA-BSA@FA-NPs treatment decreases NF-κB expression

**Fig. 8:**
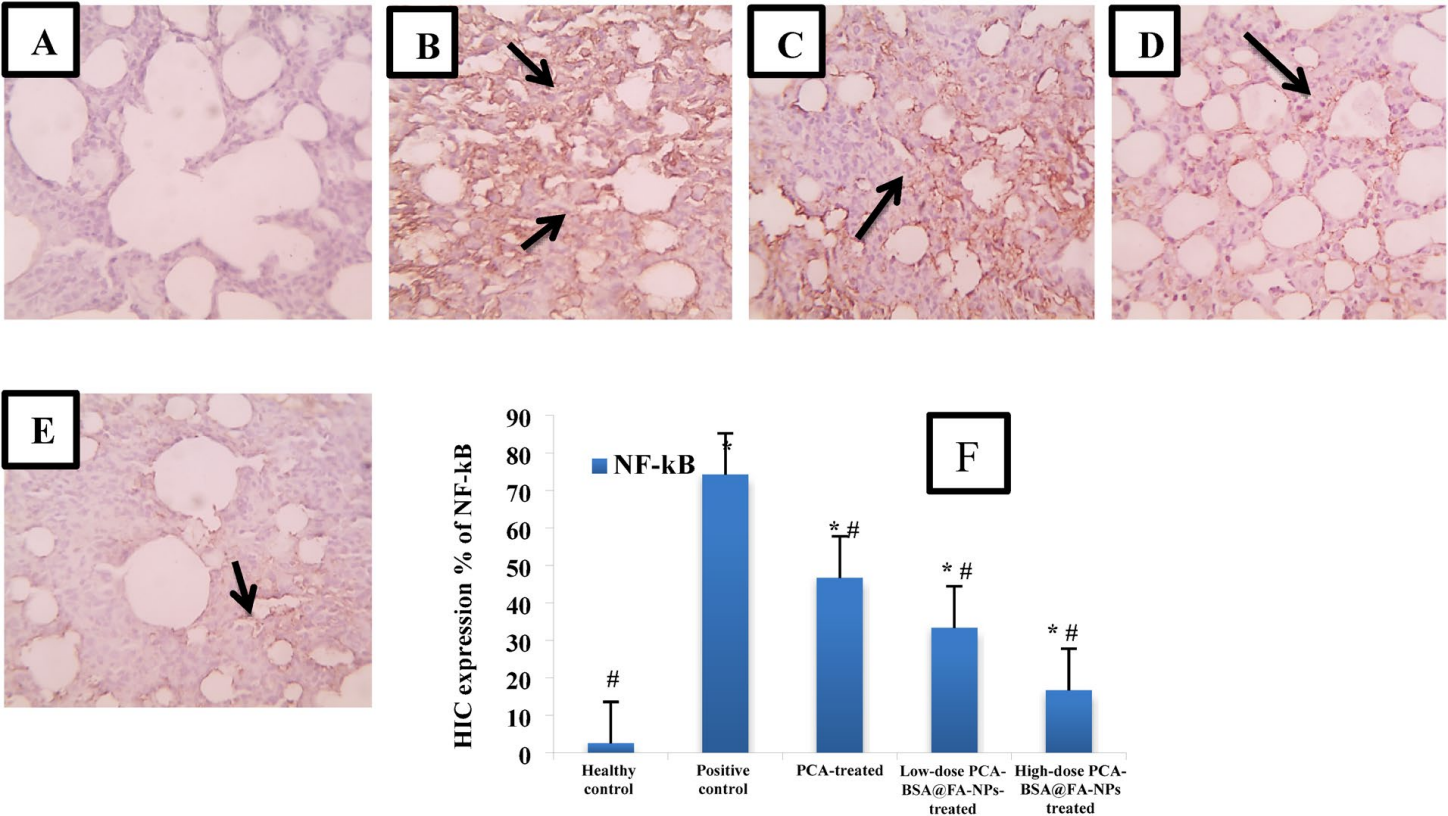
Microscopic images of NF-κB immunostained lung section; (**A**) healthy control, (**B**) positive control, (**C**) PCA-treated, (**D**) low-dose PCA-BSA@FA-NPs-treated, (**E**) high-dose PCA-BSA@FA-NPs-treated, and (**F**) Bar plot of NF-κB expression represented by mean ± SEM.* *p <* 0.05 significant compared with the healthy control group and ^#^ *p <* 0.05 significant compared with the positive control group.

NF-κB is a transcription factor essential for maintaining cellular homeostasis and responding to various stimuli. It also responds to stress, such as DNA damage, hypoxia, and oxidative stress. In non-small cell lung cancer, overexpression of NF-κB leads to increased expression of anti-apoptotic genes, helps cancer cells resist their apoptosis, and supports their continued proliferation. NF-κB protein has been linked to lung cancer progression, and its blockade in vivo significantly prevents lung cancer progression^52^. Our study established a significant increase in the NF-κB expression (14.5-fold) in lung tissue of the positive control group compared to the healthy control group (*p =* 0.0001) (Fig. 8A,B). The PCA-treated group, as well as the low-dose and high-dose PCA-BSA@FA-NPs-treated groups, exhibited significantly reduced NF-κB expression levels (1.58, 2.22, and 4.45-fold, respectively) in lung tissues when compared to the positive control group (*p =* 0.0001, for all, Fig. 8B-E). Similarly, the expressions of NF-κB were significantly reduced (1.4 and 2.79-fold, respectively) in the lung tissue of both high-dose and low-dose PCA-BSA@FA-NPs-treated groups compared to the PCA-treated group (*p =* 0.0001, for all). On the other hand, the high-dose PCA-BSA@FA-NPs-treated group showed a significant decrease in the NF-κB expression in lung tissue (1.9-fold) compared to the low-dose PCA-BSA@FA-NPs-treated group (*p =* 0.0001) (Fig. 8F). Our data indicated that treatment with a high-dose PCA-BSA@FA-NPs was more effective in inhibiting the NF-κB expression than other treated groups, demonstrating its efficiency in treating lung cancer.

#### Inhibition of FAK and MAPK expressions by PCA-BSA@FA-NPs treatment

**Fig. 9:**
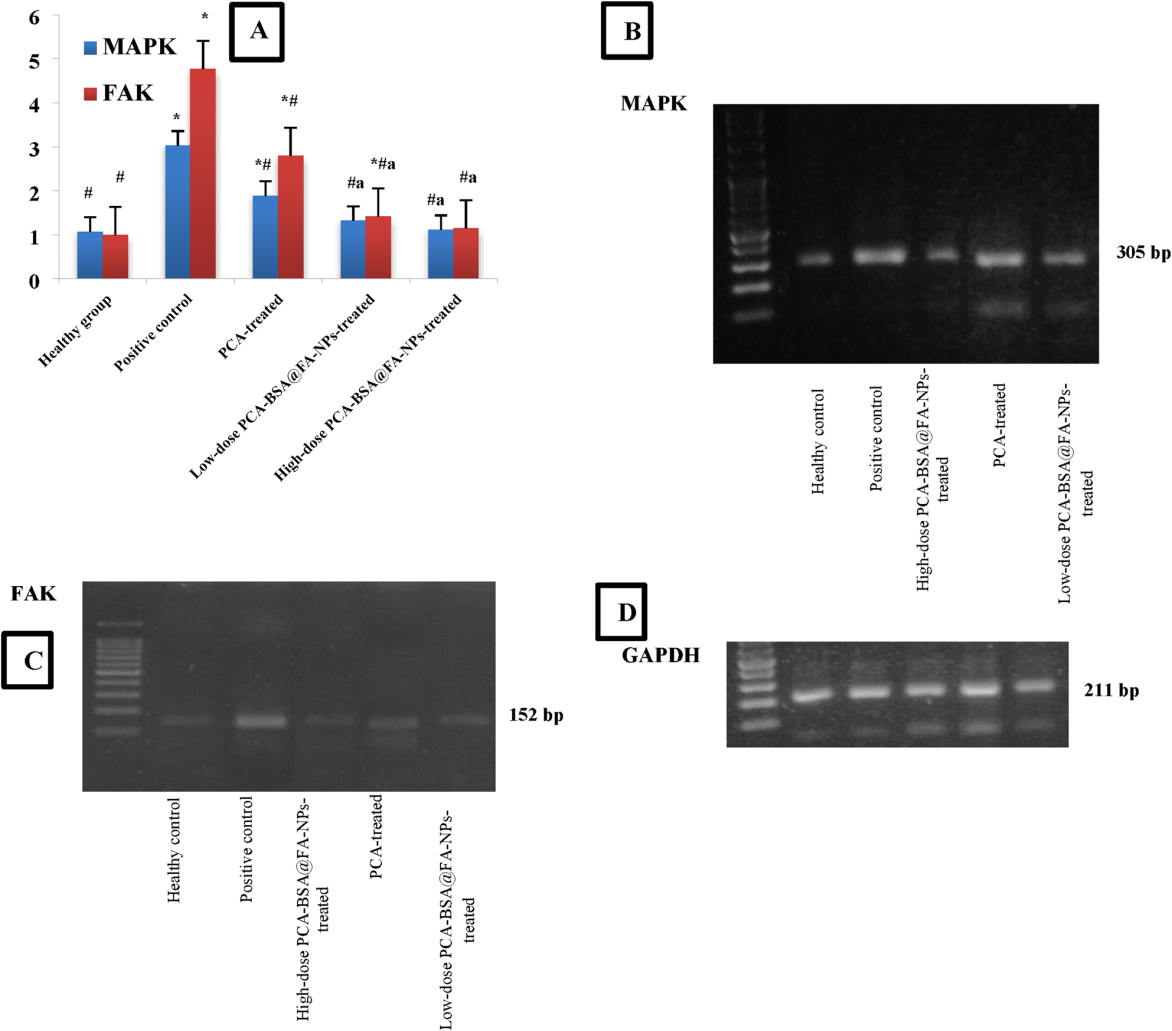
(**A**) Bar plot of MAPK and FAK represented by mean ± SEM and polymerase chain reaction-based detections of (**B**) MAPK and (**C**) FAK messenger RNAs in the lung tissues of different mice groups. (**D**) GAPDH is glyceraldehyde 3-phosphate dehydrogenase, used as a housekeeping gene. The gels were cropped from the original images, and complete gels were added in the supplementary file (Figures S1-S3). * *p <* 0.05 significant compared with the control group, ^#^ *p <* 0.05 significant compared with the Positive control group, ^a^ *p <* 0.05 significant compared with the PCA-treated group.

FAK is a non-receptor cytoplasmic protein tyrosine kinase that is widely expressed in a variety of cell types and organs. It is found in focal adhesions or sites of contact between the extracellular matrix and the actin cytoskeleton. FAK supports cell survival, migration, invasion, and metastasis by interacting with integrin and growth factor receptors. In NSCLC, FAK is overexpressed, allowing cancer cells to bypass normal growth control, leading to uncontrolled cell division, enhanced survival, increased migration, and immune suppression^53^. MAPK genes are regulatory proteins essential for cell growth, survival, proliferation, differentiation, and apoptosis, and are essential for immune responses and metabolic homeostasis. In cancer, MAPK gene expression is dysregulated by overexpression of this gene or persistent activation of MAPK pathway components, which results from loss or inhibition of phosphatases. Hyperactivation of the MAPK gene leads to the development of metastases in non-small cell lung cancer, invasion, and inhibition of apoptosis^54^. The overexpression of MAPK and FAK genes contributes to the proliferation and expansion of cancer cells in advanced tumors^4,15^. Figure 9A-D demonstrated significant increases in MAPK and FAK gene expressions in lung tissues, with increases of 2.8 and 4.77-fold, respectively, in the positive control group compared to the healthy control group (*p =* 0.0001, for both). Moreover, there were significant increases in the expressions of MAPK and FAK in the lung tissues of the PCA-treated group, with increases of 1.74 and 2.79-fold, respectively, compared to the healthy control group (*p =* 0.003 and 0.0001, respectively). However, there was no significant change in either MAPK or FAK gene expressions in lung tissues of the high-dose PCA-BSA@FA-NPs-treated group compared to the healthy control group (*p =* 1.0 and 0.76, respectively). There was also no significant change in the MAPK expression in lung tissues of the low-dose PCA-BSA@FA-NPs-treated group compared to the healthy control group (*p =* 0.68). In contrast, there was a slightly significant increase in the expression of the FAK gene (1.41-fold) in lung tissue of the low-dose PCA-BSA@FA-NPs-treated group compared to the healthy control group (*p =* 0.032). The groups treated with PCA, low-dose PCA-BSA@FA-NPs, and high-dose PCA-BSA@FA-NPs exhibited significant reductions in MAPK expression levels, with fold changes of 1.60, 2.29, and 2.72, respectively, compared to the positive control group (*p* = 0.0001 for all), indicating a potential reduction in tumor proliferation. Furthermore, these groups also showed notable decreases in FAK expression levels, with fold changes of 1.71, 3.37, and 4.16, respectively, compared to the positive control group (*p* = 0.0001 for all), correlating with reduced metastatic potential. Our results indicate that both the high-dose and low-dose PCA-BSA@FA-NPs treatment groups demonstrated significant decreases in MAPK gene expression, with fold changes of 1.43 and 1.69, respectively, compared to the PCA-treated group (*p =* 0.005 and 0.044, respectively). Also, the high-dose and low-dose PCA-BSA@FA-NPs treatment groups demonstrated significant reductions in FAK expression in lung tissues (1.97 and 2.43-fold, respectively) compared to the PCA-treated group (*p* = 0.0001 for both). Furthermore, no significant change was observed in the MAPK and FAK expressions in the lung tissue of the low-dose PCA-BSA@FA-NPs-treated group compared to the high-dose PCA-BSA@FA-NPs-treated group (*p =* 0.78 and 0.24, respectively).

FAK, MAPK, and NF-κB overexpression are crucial genes that significantly induce the growth of NSCLC cells^4,49,55,56^. In recent studies, treatment of the NSCLC with PCA has been shown to inhibit FAK, MAPK, and NF-κB gene expressions, resulting in decreased production of cytokines and growth factors, thus preventing NSCLC cell proliferation^4,5^. Our gene expression data were in line with these results, showing that PCA treatment reduced expression levels of the FAK, MAPK, and NF-κB genes, as well as decreased the expression of CD44 levels^4,5,52,57^.

## Discussion

Up to 90% of patients with lung cancer die from metastases^1^. A diagnosis of lung cancer often occurs at an advanced stage when it has spread to other parts of the body. Previous studies have revealed the potential of phenolic compounds, such as PCA, in treating lung cancer due to their ability to scavenge free radicals. However, a significant drawback is that PCA is poorly soluble^5^. In a recent study, PCA was loaded onto modified chitosan nanoparticles, enhancing its solubility and antiproliferative efficacy against A549 lung cancer cell lines^8^. In our study, PCA was encapsulated within BSA-NPs and functionalized with FA to enhance solubility and anti-lung cancer activity. The addition of target-specific ligands to nanoparticle systems has the unique ability to identify and bind to specific receptors present on specific cancer cells, increasing the targeting efficacy of the carrier and the therapeutic index of the conjugated drug. Because albumin contains carboxyl and amine groups on its surface, the covalent interaction between the desired ligands and functional groups allows for a variety of surface modifications, enrichments, and functionalization of albumin nanoparticles. Based on our desolvation method, we propose that the encapsulation of PCA occurs primarily through a rapid precipitation mechanism driven by the sudden change in solvent environment upon mixing with the antisolvent, leading to the entrapment of PCA within the forming nanoparticle matrix. Potential hydrogen bonding between PCA and the BSA-NPs matrix may further contribute to encapsulation efficiency. Researchers have discovered several receptors that are overexpressed in various types of cancer cells. Folate receptors are significantly overexpressed in various human cancer cells, including lung cancer, while being only minimally expressed in healthy tissues^58,59^. Folic acid is non-immunogenic, stable across a broad range of pH values and temperatures, and it has a unique ability to bind to folate receptors after conjugating to drug-loaded albumin nanoparticles^12,13^. Once folate has bound to the receptors, it is internalized into the cell through the endocytic pathway^42^. A slight drop in pH (to around 5) changes the conformation of the receptor, which causes folate to dissociate from the receptor and release the cytotoxic drug into the cell. Qi et al. synthesized hydrophobic drug nanoparticles and conjugated them with folic acid by solvent removal to address the nonspecific targeting and poor solubility of some hydrophobic materials^13^. For example, the hydrophobic bexarotene (BEX) was loaded onto a BSA surface and then conjugated with folic acid. The resulting nanocomposite had a diameter of approximately 200 nm, indicating a narrow size distribution. This size is quite similar to that of our nanocomposite (PCA-BSA@FA-NPs) produced in our study. In our study, the PCA-BSA@FA-NPs demonstrated an average particle size of 229 nm. Previous research has shown that nanoparticles in the range of 100–250 nm can effectively accumulate in malignant tissues due to the leakage of tumor vasculature. Considering the significant vascularization associated with lung cancers, nanoparticles of this size are likely to penetrate successfully through the cell membrane by both active targeting via folate (FA) receptors and passive targeting mechanisms^60^. Also, this size strikes a balance: large enough to avoid quick elimination by the kidneys, yet small enough to effectively infiltrate tumor tissues^61,62^. Moreover, a previous study confirmed that A549 cells demonstrated good binding to folic acid, enhancing the efficacy and specificity of BEX, promoting apoptosis, and reducing the drug’s toxicity to normal cells^17^.

Our data confirmed that the zeta potential of the PCA-BSA@FA-NPs was − 25 mV. This value, which falls between + 30 and − 30 mV, suggests that the nanoparticles are likely to be stable. Cancer cell membranes, including those of lung cancer cells, contain sialic acids, glycoproteins, and phospholipids that give them a net negative charge^63^. As a result, the negatively charged PCA-BSA@FA-NPs may interact less frequently with the cell membrane due to the repulsion of similar charges. However, active targeting methods, such as using folic acid, can enhance receptor-mediated phagocytosis and help overcome this limitation. Regardless of surface charge, the folate receptor (FR), which is widely expressed in lung cancer cells, enhances nanoparticle uptake. FA-mediated phagocytosis ensures efficient cellular internalization despite the negative zeta potential. Moreover, negatively charged nanoparticles exhibit greater stability and biocompatibility compared to positively charged ones^64^. This difference is attributed to the stronger interactions between positively charged nanoparticles and the negatively charged cell membrane, which lead to increased absorption, aggregation, and binding to serum proteins. As a result, positively charged nanoparticles may pose a higher risk of cytotoxicity and rapid clearance from the body^65^. The stability of the zeta potential values is dependent on the ionic strength of the dispersion medium^66^. PCA-BSA@FA-NPs possess a negative charge, as indicated by their negative zeta potential. Although the nanoparticles may clump together, the slight negative value helps to disperse them. Larger negative values lead to increased stability and enhanced electrostatic repulsion^67^.

In our study, BSA-NPs was used as a nanocarrier because they are highly water-soluble, non-immunogenic, non-toxic, biocompatible, biodegradable, and quickly absorbed by the body. They can also bind to various phenolic acids, such as caffeic acid and salicylic acid, in a non-covalent manner and transport them into cells easily^7,68,69^. In the current study, PCA was encapsulated within BSA nanoparticles using the desolvation method, which is the simplest approach for preparing BSA nanoparticles and encapsulating various hydrophobic phenolic acids^16,17^. Then the produced nano-compound (PCA-BSA-NPs) was conjugated with FA by carbodiimide reaction to make it more targeted to cancer cells^12,13^. In our research, the^1^H NMR spectra validated the effective creation and structural stability of BSA nanoparticles, with the broadening of peaks suggesting limited mobility. Subsequent analysis demonstrated the successful non-covalent incorporation of PCA onto BSA-NPs through hydrogen bonding, as well as the covalent attachment of Folic Acid via amide bond formation, all while maintaining the integrity of the BSA backbone^36,37^. A previous study demonstrated that BSA forms hydrogen bonds with phenolic compounds, particularly when ginsenoside CK interacts with BSA-NPs to generate BSA-CK-NPs, which was agreed with our^1^H NMR data^70^. The purpose of FA conjugation was the significant expression of the folate receptor (FR) in human cancers, while its expression remained low in healthy tissues^39–41^. This considerable variation in the FR expression pattern makes it a favorable target for selective drug delivery to cancer cells^71^. Folic acid is known for its high affinity for the folate receptor (FR)^12,19,72,73^which is overexpressed in lung cancer cells^74^. The conjugated folic acid functions as a high-affinity ligand that can selectively attach to folate receptors on the surface of cancer cells. By conjugating folic acid to PCA-BSA@FA-NPs, the nanoparticles can selectively bind to the folate receptor, facilitating receptor-mediated phagocytosis. This results in increased cellular uptake compared to non-targeted nanoparticles, making folic acid an ideal ligand for targeted drug delivery, ensuring selective interaction. Once bound, the folic acid-functionalized nanoparticles undergo receptor-mediated phagocytosis, leading to increased intracellular drug accumulation. FR was overexpressed in a wide range of human cancer cells, including the lung and brain. Folic acid has the unique capacity to attach to folate receptors^12,19,72,73^. The FR-mediated endocytosis pathway has the potential to enhance the intracellular delivery of PCA within lung cancer cells, thus improving therapeutic efficacy and reducing off-target effects on normal tissues with low FR expression. This strategy aligns directly with our goal of developing a targeted drug delivery system for cancer therapy. Furthermore, our study showed that PCA-BSA@FA-NPs was more effective and targeted lung cancer than the group treated with PCA and increased the suppression of lung cancer cell proliferation without any effect on other normal tissues. Previous research has shown that BSA-NPs are not toxic to normal human cells^75^which is consistent with our study, that BSA-NPs have no cytotoxic effect on WI38 cells. Recent research has shown that cancer cells are more susceptible to the uptake of folic acid-conjugated nanoparticles made from natural polymers such as bovine serum albumin^76,77^. Therefore, conjugating the PCA-BSA@FA nanocomposite with folic acid makes it more selective for cancer cells than for normal cells, thus reducing toxicity as it accumulates in cancer cells rather than normal cells.

Numerous studies have demonstrated that when PCA is loaded onto various nanocarrier systems, it has an increased cytotoxic effect against cancer cells^8,78,79^. PCA delivery was recently conducted using graphene oxide, hydrophobically modified chitosan, and graphene oxide-polyethylene glycol nanoparticles to enhance their susceptibility, anticancer properties, and antioxidant activity^8,78,79^. The former nanocomposite of graphene oxide–PEG loaded PCA and coated with FA demonstrated enhanced in vitro cytotoxicity against human colon cancer and liver cancer cell lines, with decreased cytotoxicity against normal cells compared to PCA^79^. These findings align with our in vitro study, showing increased cytotoxicity and reduced toxicity. However, we further conducted in vivo animal experiments, which further improved the anticancer activity as well as decreased the toxicity of our nanocomposite compared to PCA^8^. Moreover, encapsulating PCA in hydrophobically modified chitosan nanoparticles has enhanced their cytotoxicity against A549 human lung cancer, as evidenced by our in vitro study. Our study demonstrated that PCA-BSA@FA-NPs enhanced cytotoxicity against A549 cells and facilitated effective drug delivery and release. Our study on the in vivo anti-lung cancer activity of PCA-BSA@FA-NPs yielded unique results, as previous studies only focused on in vitro investigations. We evaluated the effects of PCA-BSA@FA-NPs in treating lung cancer while also assessing organ toxicity.

In our study, we used BSA-NPs as a good carrier because they are non-immunogenic, biocompatible, and biodegradable^69^. Therefore, PCA was encapsulated within BSA-NPs, then conjugated with FA and measured for bioactivity. Several studies have established the enhanced cytotoxic activity of PCA against cancer cells after being loaded on different nanocarrier systems^8,22,78–80^. In our study, the resulting PCA-BSA@FA-NPs were more soluble in water than PCA. PCA-BSA@FA-NPs showed a cytotoxic effect against A549 and also less toxicity against WI38 cells compared to PCA and DOX. Although PCA-BSA@FA-NPs has a higher IC_50_ value compared to DOX when tested against A549 cells, indicating that DOX is more effective in this context, DOX demonstrates a significantly lower IC_50_ against WI38 cells compared to PCA-BSA@FA-NPs. This suggests that DOX exhibits greater toxicity towards normal cells. DOX generates free radicals that can damage proteins and DNA in both cancer and normal cells^81^. In contrast, PCA-BSA@FA-NPs are proposed to act by scavenging the excess free radicals produced during cancer metabolism. This helps to stop cancer proliferation while simultaneously protecting normal cells from further damage caused by these free radicals. Previous studies have shown that PCA exhibits antioxidant activity by scavenging free radicals^82–84^. This is supported by PCA’s ability to scavenge DPPH radicals and the increased activities of SOD and catalase^5,24^. Our findings indicate that PCA can scavenge ROS via scavenging the DPPH radical. Additionally, PCA-BSA@FA-NPs had a higher capacity to scavenge DPPH radicals than PCA alone. Our previous study also demonstrated improved antioxidant, SOD-like activity, catalytic activity, and in vitro anticancer activities of PCA after loading it onto BSA-NPs (PCA-BSA-NPs) compared to PCA^24^. Herein, our findings indicate that PCA-BSA-NPs and PCA-BSA@FA-NPs had greater SOD activity compared to PCA. PCA-BSA@FA-NPs demonstrate superior CAT activity compared to PCA and PCA-BSA-NPs, demonstrating the efficacy of PCA-BSA@FA-NPs as a powerful antioxidant.

Our drug release study results showed that PCA-BSA@FA-NPs release PCA more effectively in the cancer environment than in the physiological environment. This supports our hypothesis that the PCA-BSA@FA-NPs is biologically effective and target cancer cells than normal cells. The safety of PCA-BSA@FA-NPs on the vital organs was assessed in the treated control mice with low and high doses of the nano-formulation, as well as PCA, and compared with the healthy control group. No toxicity was observed in the liver and kidneys in the low and high PCA-BSA@FA-NPs dose groups. Although the differences in creatinine levels between the control and treatment groups were small, the increased creatinine levels in the PCA control group may indicate early renal effects or temporary changes in kidney function. It may also indicate a more pronounced effect on kidney function than in the PCA-BSA@FA-NPs control groups, suggesting the overall safety of PCA-BSA@FA-NPs.

Reactive oxygen species (ROS) are very high in cancer cells due to increased cyclooxygenase and metabolic activity^46^. ROS damages DNA, tissues, and organs. The function of antioxidant enzymes is to neutralize different forms of ROS. However, when these levels rise as a result of cancer, the activity of these enzymes, including SOD, and CAT decreases, while oxidative stress marker MDA increases^46,47^. The clinical importance of antioxidants in lung cancer treatment stems from the association of lung cancer with increased oxidative stress, which leads to DNA damage, inflammation, and tumor progression. Antioxidants help neutralize free radicals, which may slow cancer growth, reduce treatment side effects, and protect cells from oxidative damage. Antioxidants may help prevent the progression of lung cancer by protecting against it^47,48,85^. To prevent cell damage, SOD scavenges superoxide anion, while CAT can scavenge intracellular hydrogen peroxide and lessen oxidative cell damage^46^. When ROS damages lipids in cell membranes, it produces a reactive byproduct known as MDA. MDA is frequently employed as a biomarker for cellular damage and oxidative stress^47,48^. The decrease in SOD and CAT levels, and raised MDA levels, were observed in lung cancer^46–48^. Therefore, antioxidant enzymes (SOD and CAT) as well as MDA were measured in all groups before and after treatment. In consistency with our results, previous studies have shown that PCA can decrease the oxidative stress marker MDA and scavenge free radicals by increasing the levels of SOD, CAT^5,43^. In our study, the PCA-BSA@FA-NPs demonstrated an enhanced ability to reduce oxidative stress. This was evidenced by a significant increase in the levels of SOD and CAT, along with a decrease in MDA levels, compared to PCA alone.

The CD44 protein increases its expression on the cancer cell’s surface. CD44 plays a crucial role in the progression of cancer. It contributes to processes such as metastasis, invasion, adhesion, and angiogenesis^49–51^. FAK is a non-receptor cytoplasmic protein tyrosine kinase that is widely expressed in a variety of cell types and organs. It is found in focal adhesions, or sites of contact between the extracellular matrix and the actin cytoskeleton. FAK supports cell survival, migration, invasion, and metastasis by interacting with integrin and growth factor receptors. In NSCLC, FAK is overexpressed, allowing cancer cells to bypass normal growth control, leading to uncontrolled cell division, enhanced survival, increased migration, and immune suppression^53^. MAPK genes are regulatory proteins essential for cell growth, survival, proliferation, differentiation, and apoptosis, and are essential for immune responses and metabolic homeostasis. In cancer, MAPK gene expression is dysregulated by overexpression of this gene or persistent activation of MAPK pathway components, which results from loss or inhibition of phosphatases. Hyperactivation of the MAPK gene leads to the development of metastases in non-small cell lung cancer, invasion, and inhibition of apoptosis^54^. NF-κB is a transcription factor essential for maintaining cellular homeostasis and responding to various stimuli. It also responds to stress, such as DNA damage, hypoxia, and oxidative stress. In non-small cell lung cancer, overexpression of NF-κB leads to increased expression of anti-apoptotic genes, helps cancer cells resist apoptosis, and supports their continued proliferation^86^. FAK, MAPK, and NF-κB overexpression are crucial genes that significantly induce the growth of NSCLC cells^4,49,55,56^. Tumor development, survival, and metastasis are significantly influenced by the signaling pathways of NF-κB, MAPK, and FAK. A crucial transcription factor, NF-κB controls genes related to angiogenesis, inflammation, and apoptosis inhibition. Its overexpression increases survival genes (including Bcl-2 and Bcl-xL) and encourages the growth of tumor cells. Additionally, it increases vascular endothelial growth factor, which promotes angiogenesis. Additionally, it makes tumors more aggressive and more likely to spread. Reduced NF-κB expression in treated groups may therefore be a sign of both therapeutic success and tumor growth suppression^87^. In our study, PCA-BSA@FA-NPs treatment significantly reduced NF-κB levels by 2.79-fold in the lung tissue compared to the PCA-treated group, indicating its effectiveness in inhibiting lung cancer proliferation.

Since cell survival and proliferation are encouraged by the ERK/MAPK pathway, MAPK contributes to the advancement of cancer. Through the activation of survival signals, its overexpression causes unchecked cell division and increases resistance to treatment. By activating matrix metalloproteinases (MMPs), it also encourages metastasis. Therefore, it may be a sign of enhanced apoptosis and suppression of proliferation if MAPK levels fall in the therapy group^88^.

Cell adhesion, migration, and invasion all depend on FAK. Aggressive cancers possess higher FAK levels, which promote metastasis and epithelial-mesenchymal transition (EMT). The mobility of cancer cells is improved by increased FAK expression, which encourages distant metastasis. Consequently, a lower chance of metastasis is indicated by lowered FAK levels in response to treatment^15^. In recent studies, treatment of NSCLC with PCA has been shown to inhibit FAK, MAPK, and NF-κB gene expressions, resulting in decreased production of cytokines and growth factors, thus preventing NSCLC cell proliferation^4,5^. Our data showed that treatments with PCA-BSA@FA-NPs were more effective than PCA alone. PCA-BSA@FA-NPs treatment resulted in a reduced tumor area percentage, indicating a decrease in lung cancer proliferation. This effect may have occurred through the modulation of the gene expressions of FAK, MAPK, and NF-κB. Our gene expression data were in line with these results, showing that PCA treatment reduced expression levels of the FAK, MAPK, and NF-κB genes as well as decreased the expression of CD44 levels^4,5,52,57^. In addition, when PCA was encapsulated within BSA-NPs and conjugated with FA, its ability to reduce the expression of these genes increased, which enhanced its ability to suppress lung cancer cells. PCA-BSA@FA-NPs have small particles size (229 nm). This small size may have enhanced PCA solubility and penetration into cancer cells, allowing them to neutralize free radicals within the cells. This appeared in the increased SOD and CAT levels, along with decreased MDA levels. As a result, vital organs were protected from oxidative damage, and the proliferation of lung cancer cells was inhibited. Moreover, PCA-BSA@FA-NPs treatment led to reduced CD44 levels, which may inhibit lung cancer angiogenesis and metastasis. The inhibited angiogenesis led to proliferation inhibition, which appeared in the lower expression of FAK, MAPK, and NF-κB genes.

## Conclusion

In this study, PCA was encapsulated within BSA-NPs, resulting PCA-BSA@FA-NPs nanocomposite with 229 nm in size. Our results show that PCA-BSA@FA-NPs has anticancer activity against the A549 cell line with lower toxicity to normal cells compared to PCA. Additionally, PCA-BSA@FA-NPs exhibited greater antioxidant activity than PCA, similar to vitamin C. Our in vivo study demonstrated the ability of PCA-BSA@FA-NPs to neutralize free radicals by increasing SOD and CAT levels and decreasing MDA levels in lung tissues. PCA-BSA@FA-NPs could enter the cancer cells through the FR-facilitate endocytosis. After entrance, it inhibits the expression of NF-kB, FAK, and MAPK genes, as well as the expression of the CD44 protein in lung cancer tissues, indicating its ability to inhibit lung cancer growth. Our study also showed that there is no evidence that PCA-BSA@FA-NPs are toxic to vital organs, unlike PCA, which causes an increase in kidney function. Therefore, the encapsulation of PCA into folate-conjugated albumin nanoparticles enhances its efficacy and targeting for cancer cells. This improved the ability of PCA-BSA@FA-NPs to inhibit tumor growth and reduce their toxicity during administration. In conclusion, PCA-BSA@FA-NPs may represent a promising strategy for delivering PCA to tumors in a targeted manner. However, many experiments and thorough analyses are still required to determine the precise anti-cancer mechanism of the nanocomposite (PCA-BSA@FA-NPs) and to confirm its safety and effectiveness in targeting lung cancer for human application. In addition to ensuring it does not harm vital organs, further studies should also be conducted to evaluate its safety.

In our further research, we intended to evaluate the in vivo behavior of PCA-BSA@FA-NPs such as the circulation half-life of the nanoparticles, their distribution to the tumor site and other organs, the rate of PCA release in vivo, stability of the nanoparticles in the bloodstream, their ability to reach the target site, and their overall bioavailability, and the elimination pathways.

## Electronic supplementary material

Below is the link to the electronic supplementary material.

Supplementary Material 1

## Data Availability

The datasets used and analyzed during this study are available from the corresponding author upon reasonable request.
