# Supplementary material for: Novel protocatechuic acid encapsulated bovine serum albumin functionalized folic acid nanoparticles for targeted therapy in urethane-induced lung cancer model
Source: Sci Rep. 2025 Jul 16;15:25793. doi: 10.1038/s41598-025-08465-6 (PMC12267497; doi:10.1038/s41598-025-08465-6)
Supplement: Supplementary file 1 — Supplementary Material 1 [file 41598_2025_8465_MOESM1_ESM.docx]

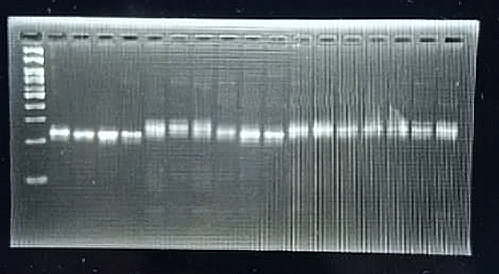


MAPK gene 305 bp

**Con.**

**PRT LDT HDT**

**PC**

**PC**

**LDT HDT PRT Con.**

**Ladder**


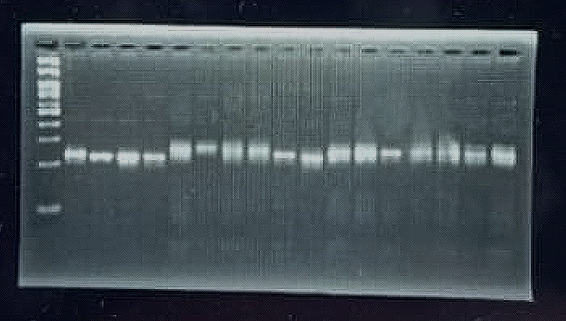


MAPK gene 305 bp

**LDT HDT PRT Con. PC**

**Ladder**

**Figure S1.** Agarose gel electrophoresis showed replication of PCR products for the MAPK gene in all studied groups. HDT: High-dose PCA-BSA@FA-NPs-treated, LDT: Low-dose PCA-BSA@FA-NPs-treated, PRT: PCA-treated, Con.: Healthy control group, and PC: positive control group


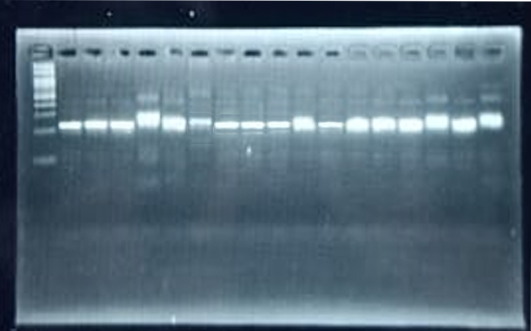


FAK gene 152 bp

**PRT LDT HDT PC Con. PC**

**HDT LDT PRT PC PC Con.**

**Ladder**


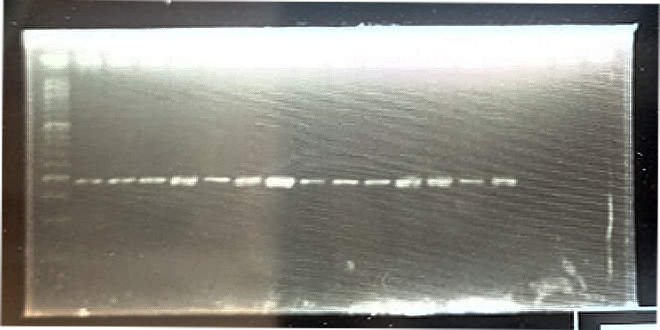


FAK gene 152 bp

**HDT LDT PRT PC PC Con.**

**HDT LDT LDT PRT Con. PC PC**

**Ladder**

**Figure S2.** Agarose gel electrophoresis showed replication of PCR products for the FAK gene in all studied groups. HDT: High-dose PCA-BSA@FA-NPs-treated, LDT: Low-dose PCA-BSA@FA-NPs-treated, PRT: PCA-treated, Con.: Healthy control group, and PC: positive control group


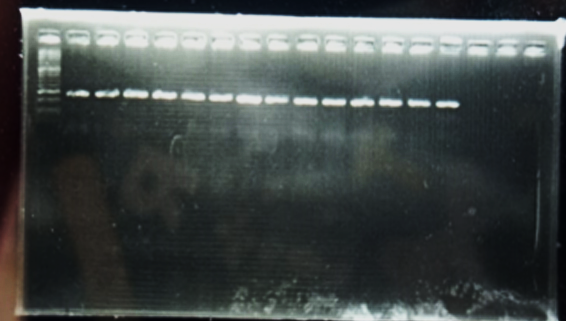


GAPDH gene 211 bp

**Figure S3.** Agarose gel electrophoresis showed replication of PCR products for the GAPDH gene in all studied groups.
